# A look back on how far to walk: Systematic review and meta-analysis of physical access to skilled care for childbirth in Sub-Saharan Africa

**DOI:** 10.1371/journal.pone.0184432

**Published:** 2017-09-14

**Authors:** Kerry L. M. Wong, Lenka Benova, Oona M. R. Campbell

**Affiliations:** Department of Infectious Disease Epidemiology, Faculty of Epidemiology & Population Health, London School of Hygiene & Tropical Medicine, London, United Kingdom; National Academy of Medical Sciences, NEPAL

## Abstract

**Objectives:**

To (i) summarize the methods undertaken to measure physical accessibility as the spatial separation between women and health services, and (ii) establish the extent to which distance to skilled care for childbirth affects utilization in Sub-Saharan Africa.

**Method:**

We defined spatial separation as the distance/travel time between women and skilled care services. The use of skilled care at birth referred to either the location or attendant of childbirth. The main criterion for inclusion was any quantification of the relationship between spatial separation and use of skilled care at birth. The approaches undertaken to measure distance/travel time were summarized in a narrative format. We obtained pooled adjusted odds ratios (aOR) from studies that controlled for financial means, education and (perceived) need of care in a meta-analysis.

**Results:**

57 articles were included (40 studied distance and 25 travel time), in which distance/travel time were found predominately self-reported or estimated in a geographic information system based on geographic coordinates. Approaches of distance/travel time measurement were generally poorly detailed, especially for self-reported data. Crucial features such as start point of origin and the mode of transportation for travel time were most often unspecified. Meta-analysis showed that increased distance to maternity care had an inverse association with utilization (n = 10, pooled aOR = 0.90/1km, 95%CI = 0.85–0.94). Distance from a hospital for rural women showed an even more pronounced effect on utilization (n = 2, pooled aOR = 0.58/1km increase, 95%CI = 0.31,1.09). The effect of spatial separation appears to level off beyond critical point when utilization was generally low.

**Conclusion:**

Although the reporting and measurements of spatial separation in low-resource settings needs further development, we found evidence that a lack of geographic access impedes use. Utilization is conditioned on access, researchers and policy makers should therefore prioritize quality data for the evidence-base to ensure that women everywhere have the potential to access obstetric care.

## Introduction

Place forms part of the construct of social and physical interactions and resources, which influence health and wellbeing [1,2]. Geocoding, residential mobility, record linkage and data integration, spatial cluster detection, small area estimation and Bayesian applications mapping are examples of methodologies used to investigate health-related issues from a geographic perspective [3–5]. Increasingly, spatial thinking and geographic information system (GIS) tools are being applied to different public health and epidemiological topics, including global maternal, newborn and child health [6–8].

In the post-2015 era, the international communities continue to prioritize reducing preventable maternal and newborn deaths in low- and middle-income countries (LMICs) [1,5]. As part of the effort to achieve Sustainable Development Goal (SDG) 3.1 of reducing global maternal mortality ratio (MMR) to <70 per 100,000 live births by 2030, researchers and policy makers call specifically for equitable, within-country improvement [1,5]. The United Nations Sustainable Development Summit held in 2015 promoted mapping and other GIS tools be used to address localized health inequalities, targeting the hard-to-reach population at the subnational scale [9].

Accounting for a mere 13% of the global population, Sub-Saharan Africa (SAA) is home to two thirds of women who died of maternal causes globally [10]. Most maternal deaths in SSA are preventable [11]. The World Health Organization (WHO) has advocated for skilled care at every birth as one of the main strategies to ensure safe motherhood and combat maternal mortality [12]. In much of the region, however, fewer than half of all women received skilled care at birth [13]. Utilization is a complex issue driven by different personal, behavioural and cultural factors [14,15]. It has also been argued that universal healthcare utilization (including that of delivery care) is conditioned on everyone having potential access to health services [16–18]. Potential access has three system-level dimensions—services must be physically accessible, financially affordable and acceptable to those who require care if universal coverage is to be attained [18].

The effect of each of the three dimensions on usage of skilled care at birth in SSA and other resource-limited settings have been discussed in previous literature reviews. The most recent systematic review, found physical distance between health facilities and service user’s residence to be one of the most significant barriers [19], confirming findings from earlier reviews [14,15]. Ongoing global attention on the SDG, coupled with technological advancements in GIS tools have driven researchers to better quantify and examine the impact of physical accessibility, mostly capturing it as the spatial separation between women and health services [3]. This calls for an effort to synthesize available evidence to appraise the different measurement approaches used, reassess spatial separation between women and available health services, and to better understand the effect of increased spatial separation on skilled delivery care utilization in SSA.

The objectives of this systematic review are to (i) provide an overview of the approaches undertaken to measure spatial separation (as distance and travel time) between women and health services and (ii) establish to extent to which spatial separation deters utilization of skilled care for childbirth in Sub-Saharan Africa using existing quantitative evidence.

## Methods

### Review question and search strategy

A systematic review was conducted to search, summarize and synthesize evidence using five databases—Medline, Embase, Global Health, Africa Wide Information and Popline. The search was performed in March 2016 for materials published between January 1986 and February 2016. The year cut-off of 1986 was chosen as it was the time when activists and professionals first started mobilizing around safe motherhood [20]. Using both MeSH terms and free-text terms, the search was designed to identify articles covering all three themes—(i) SSA, (ii) distance or travel time and (iii) utilization of skilled care at birth. A sample of search terms used is given in Box 1 and the complete search strategies for each database used is given in S1 Table.

**Box 1. Keywords and phrases for searching**

(i) **Sub-Saharan Africa**
  Individual country name; Sub-Saharan Africa; Africa South of the Sahara; multi-country; cross-culture; developing countries
(ii) **Geographic access**
  Geospatial; geographic information system; kilometre; physical access; distance; travel; transport
(iii) **Skilled care at birth**
  Facility birth; hospital birth; skilled birth attendant; traditional birth attendant; trained assistant

### Selection criteria, data extraction and study quality assessment

We removed duplicated records. Abstracts of unique studies identified were screened and discarded if they were irrelevant to the study question. We included studies that quantified the relationship between the magnitude of distance or travel time and women’s actual use of skilled care at birth. Studies that only reported women’s plan to use skilled care for future childbirths and studies that only reported women’s opinion or perception on physical access as a reason for where or with whom to give birth were excluded. Reference lists of included papers were reviewed to identify additional studies, which were subjected to the same review process.

Descriptive information abstracted from the final list of included studies were study design, study objective, study sample and data source. We extracted studies’ mean distance/travel time. If the mean was not provided, we approximated it as the product of the midpoint of each category (midpoint of last category with an unspecified upper bound is given as the lower bound + 0.5 × width of second last category). We also extracted the mean level of skilled care at birth. Information on the approaches taken to measure exposure (distance and travel time) were also extracted; this included the data collection method and the definition of spatial separation—start point of origin, ending destination and type of line distance (straight or road network) or the mode of transportation for travel time (e.g., walking, driving). We also abstracted studies’ outcome definition of skilled care at birth (delivery location and/or attendant). Crude and adjusted parameter estimates of effect sizes, and the confounding variables used in adjusted analysis (if available) were abstracted.

The quality assessment of the studies was carried out using a modified version of the National Heart, Lung and Blood Institute’s Quality Assessment Tool for Observational Cohort and Cross-Sectional Studies [21]. We assessed sample representativeness, sources of selection and location bias, whether exposure had been clearly defined and accurately measured. The assessment of outcome quality was based on whether the type of delivery location/attendant considered to be skilled and unskilled was clearly defined and accurately measured. We also recognize that some determinants of skilled facility-based delivery in SSA identified in previous literature reviews may confound the association between spatial separation and use of skilled care. Specifically, low financial means and low education both influence one’s place of residence and healthcare use, and an individual in a more serious (self-assessed or otherwise informed) health situation might be more inclined to use healthcare given the same distance [22]. Therefore, multivariate analyses were considered as adequately-adjusted if affordability, education and need (or perceived need) for skilled care at birth were controlled for. We considered adjustment for parity, pregnancy complications, previous stillbirths, among others, as suitable proxy for (perceived) need for skilled care at birth.

All studies were reviewed by KLMW, and a 5% sample of studies was independently reviewed by LB and OMRC. Conflicts were resolved by discussions among KLMW, LB and OMRC.

### Synthesis of data and meta-analysis

We created typology of distance and travel time measurement on the basis of data collection method and the definition used. According to the type of end location, studies results from adequately-adjusted analyses were first presented in a narrative format. The use of any nearest health facility (HF) regardless of its capacity to provide maternity care may bias women’s true separation from skilled care provision. Meta-analysis is therefore restricted to adequately-adjusted results that defined the destination as a location with maternity care provision. To combine effect estimates referring to both a continuous variable and a categorical variable, results of the latter—where three or more levels were used—were converted with trend estimation technique proposed by Greenland and Longnecked (trend estimation is not possible for dichotomous comparisons) [23]. Estimated trends and adjusted odds ratios (AORs) of other studies that considered distance as a continuous variable were pooled in meta-analyses to test the effect of physical accessibility on the use of skilled care at birth. We were not able to retrieve unreported insignificant adjusted effects from some qualifying studies from the corresponding authors, in which case we included the unadjusted results that were presented to minimize biases in the pooled estimate [24].

## Results

### Study identification

**Fig 1:**
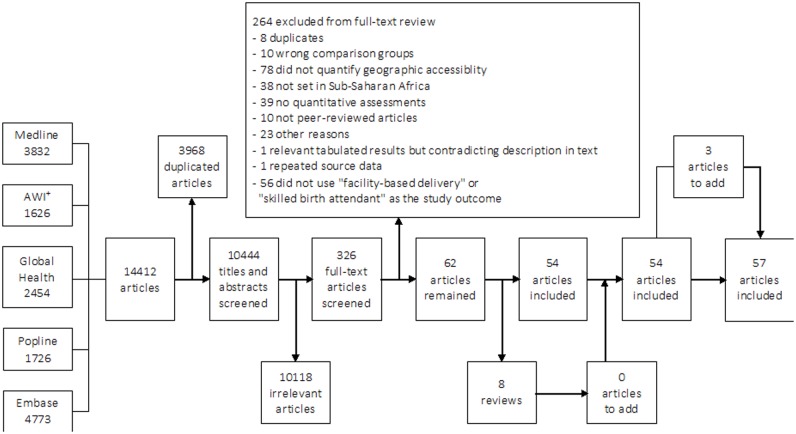
Flow chart of study selection for inclusion in the systematic review. ^+^AWI = Africa Wide Information.

Initial search results obtained from the databases totalled 14,412 articles. After de-duplication, 10,444 remained, of which 10,118 were discarded for irrelevance following title and abstract screening. The 326 potentially relevant articles were retained for full-text review, and 57 met the inclusion criteria. A flow diagram detailing the number of studies screened and assessed for eligibility, with reasons for exclusion at each stage of the review process, is provided in Fig 1.

### Characteristics of included studies

**Fig 2:**
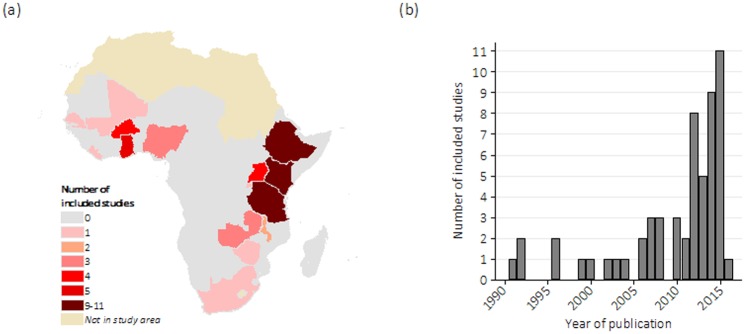
(a) Geographic coverage^+^ and (b) year of publication of 57 included studies. ^+^Reprinted from Map Maker Limited under a CC BY license, with permission from Map Maker Limited, original copyright 2017.

Out of 57 studies, 30 were conducted in Tanzania, Ethiopia and Kenya (S2 Table). No studies were found in 33 of 48 countries in SSA [25], including, for instance, the whole sub-region of Central Africa (Fig 2(a)). Eight studies (14%) were conducted at a national scale. The oldest identified article was published in 1991, but over two thirds of all included studies emerged since 2010 (Fig 2(b)). All included studies were cross-sectional. De Groot et al. 1993 [26] interviewed women as they attended a health facility (HF) for childbirth during the study period; the remaining 56 studies were retrospective. Esimai et al. 2007 [27] was set in urban Nigeria, 18 studies were set in both urban (or semi-urban) and rural areas, 34 studies were in rural area only, one was in a conflict context in Uganda and three did not provide clear information. Fourteen of 57 (25%) studies examined distance or travel time as a primary objective. The numbers of distance and travel time measurements were 40 and 25, including eight studies that measured both.

The results of the quality assessments are summarized in Table 1. Selection bias was identified in 28 studies. Particularly, sample selection in 14 of these 28 studies were confined to the easier-to-reach subpopulations, such as women living inside the catchment area of a HF. The other 14 study samples were drawn from existing health-seekers or registered residents who might have higher tendency to utilize skilled care.

**Table 1:** Quality assessment of 57 included studies.

	Yes	No	Unclear
**Potential selection bias (n = 57)**			
Study sample subject to greater physical accessibility (location bias)	14 (25%)	43 (75%)	0 (0%)
Study sample more likely to delivery with skilled care	14 (25%)	43 (75%)	0 (0%)
**Study outcome (n = 57)**			
Self-reported data of type of care used	54 (95%)	2 (4%)	1 (2%)
Clearly defined as source of skilled obstetric care	26 (46%)	29 (51%)	2 (4%)
**Adjustment for potential confounder (n = 57)**			
Affordability or financial means	37 (65%)	20 (35%)	0 (0%)
Education	41 (72%)	16 (28%)	0 (0%)
Need or perceived need of skilled care at birth	37 (65%)	20 (35%)	0 (0%)
All of the above	29 (51%)	28 (49%)	0 (0%)
**Study exposure—measurements of distance (n = 40)**^			
Self-reported data only	22 (55%)	14 (35%)	4 (10%)
Clearly defined with start and end points and distance/transportation type	12 (30%)	28 (70%)	0 (0%)
Defined as starting from women’s home and ending at a specified facility	2 (5%)	10 (25%)	28 (70%)
**Study exposure—measurements of travel time (n = 25)**^			
Self-reported data only	22 (88%)	2 (8%)	1 (4%)
Clearly defined with start and end points and distance/transportation type	3 (12%)	22 (88%)	0 (0%)
Defined as starting from women’s home and ending at a specified facility	1 (4%)	2 (8%)	22 (88%)
**High-quality study (n = 57)**			
Sample selection unlikely to be biased, well-defined exposure and outcome and adequately adjusted for all three potential confounders	0 (0%)	57 (100%)	0 (0%)

^The numbers of distance and travel time measurements are 40 and 25, including eight studies that measured both.

Outcome (self-reported in 95% of the included studies) was considered clearly and well defined in 26 (46%) studies. The rest were unclear or prone to misidentifying the use of skilled care at birth by, for instance, not considering non-home births and births at HFs of any level as unskilled. Quality assessment of exposure measurement as well as confounding is presented in the following section together with examination of measurement approaches and effect of exposure. Overall, we did not find a high quality study that had an unbiased sample, a well-defined exposure and outcome, and adequate adjustment for all of affordability, education and (perceived) need for skilled care at birth.

### Measuring distance

#### Quality of distance measurements

Among 40 studies with distance measurements, 12 were clearly defined with the start and end points as well as the distance type as straight-line or one along road (Table 1). Two of these 12 were well-defined as ending at certain specified locations likely to be able to offer labouring women skilled delivery care.

#### Typology of distance measurements

Table 2(a) presented the typology of the 40 studies with distance measurements. Twenty-two (55%) studies collected self-reported data only; 9 of which measured distance to any nearest HF regardless of its capacity to provide maternity care, while another 13 used one or more specified HFs as the end. All 22 studies conducted a survey and interviewed women about their distance to healthcare as part of a structured questionnaire; six of which stated a start point of origin (home or women’s communities) and none of which detailed whether distance was straight-line or travelled along a road. The three studies with unclear data collection methods also provided little information on how distance was defined (Table 2a).

**Table 2:** Typology of (a) measurements of distance in 40 studies and (b) measurements of travel time in 25 studies.

**(a)**	**Data collection method**																**Total**
**Endpoint**	**Self-reported**				**Measured or estimated**				**Both**				**Unclear**				
**Nearest health facility only**	**Line type**	**Start point**			**Line type**	**Start point**							**Line type**	**Start point**			12
		**Home**	**Community**	**Unclear**		**Home**	**Community**	**Unclear**						**Home**	**Community**	**Unclear**	
	**Unclear**	1	1	7	**Unclear**								**Unclear**		1		
	**Straight line**				**Straight line**	1							**Straight line**				
	**Road network**				**Road network**			1					**Road network**				
	**Both**				**Both**								**Both**				
**Health facility equipped to provide skilled care for childbirth**	**Line type**	**Start point**			**Line type**	**Start point**			**Line type**	**Start point**			**Line type**	**Start point**			
		**Home**	**Community**	**Unclear**		**Home**	**Community**	**Unclear**		Home	Community	Unclear		**Home**	**Community**	**Unclear**	
	**Unclear**	2	2	9	**Unclear**		1	1	**Unclear**				**Unclear**		1		
	**Straight line**				**Straight line**		3		**Straight line**		1		**Straight line**				
	**Road network**				**Road network**		2		**Road network**				**Road network**				
	**Both**				**Both**		1		**Both**				**Both**				
**Both nearest & equipped health facility**					**Line type**	**Start point**							**Line type**	**Start point**			5
						**Home**	**Community**	**Unclear**						**Home**	**Community**	**Unclear**	
					**Unclear**								**Unclear**			1	
					**Straight line**	2	1						**Straight line**				
					**Road network**		1						**Road network**				
					**Both**								**Both**				
**Total**	22				14				1				3				40
**(b)**	**Data collection method**																
**Endpoint**	**Self-reported**				**Measured or estimated**				**Both**				**Unclear**				
**Nearest health facility only**	**Line type**	**Start point**															13
		**Home**	**Community**	**Unclear**													
	**Unclear**			4													
	**Walking**		1	8													
	**Mechanized**																
	**Both**																
**Health facility equipped to provide skilled care for childbirth**	**Line type**	**Start point**			**Line type**	**Start point**											12
		**Home**	**Community**	**Unclear**		**Home**	**Community**	**Unclear**									
	**Unclear**			6	**Unclear**												
	**Walking**	1		3	**Walking**												
	**Mechanized**				**Mechanized**		1										
	**Both**				**Both**		1										
**Total**	23				2				0				0				25

HF = health facility

In the remaining 15 studies which did not use self-reported exposures, distance was measured or estimated using geographic data, including one (De Groot et al. 1991 [26]) that interviewed women attending a HF for childbirth about their distance from home village, whilst address and other data for women with non-facility births were calculated using local census data. Among the other 14 studies, Nwakoby 1992 [28] measured distance directly on a printed map; and Kenny et al. 2015 [29] tracked distance with a handheld positioning device during field workers’ travels to the communities for household interviews. The rest integrated geolocated data of women’s home or communities and a complete listing of HFs of the study area in a GIS. Seven of these then estimated straight-line distance from geospatial coordinates; and four others—Mills et al. 2008 [30], Joharifard et al. 2012 [31], Nesbitt et al. 2014 [32] and Johnson et al. 2015 [33]–estimated road network distance by further adding a shape file of vector data (a file to store geometric data of geographic features represented by points, lines or shapes) of the study area’s road systems in their GISs.

#### Effects of distance on skilled care at birth

**Fig 3:**
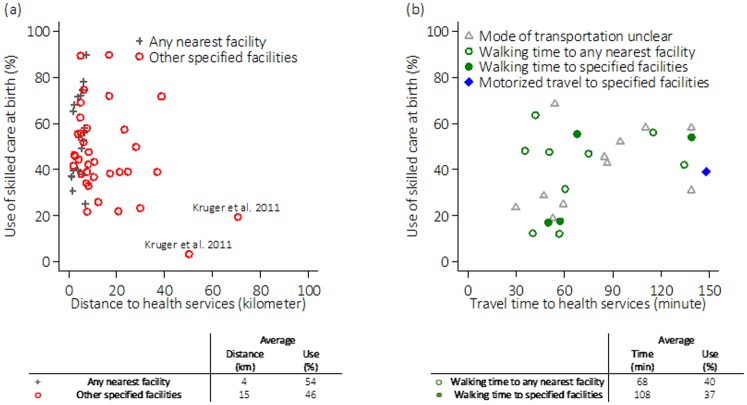
Summary of included studies’ mean levels of use of skilled care at birth against (a) average distance to health services in kilometres (km) and (b) average travel time to health services in minutes (min).

The effects of distance across the 40 studies were first summarized by their sample-level mean proportions of skilled care at birth and mean distances (Fig 3(a)). Multi-site studies, or studies reporting distances to more than one end points are represented separately. Fig 3(a) suggested that there was little to no difference in use as distance from specified HFs changed within the 40km-bound across all included studies. As distance from specified HFs increased beyond this threshold, however, a drop in skilled care utilization was observed, suggesting certain non-linear effect. This pattern appeared to be driven heavily by only one multi-site study (Kruger et al. 2011 [34]), however, another two studies stratified their study subjects into living “closer” and “further away” from health services (Hounton et al. 2008 [35] and Mwaliko et al. 2014 [36]) and their results were in line with this observed non-linearity, as they both found a negative effect of distance on use only among women who lived “closer”.

**Fig 4:**
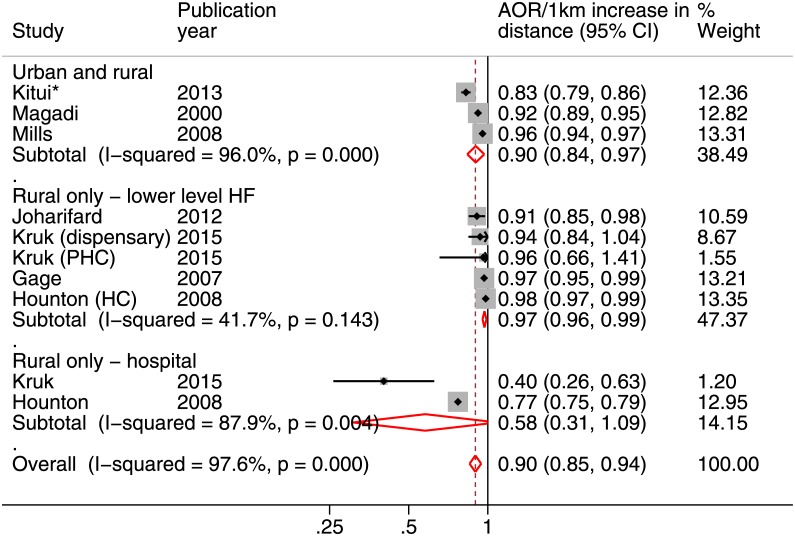
Forest plot showing the adjusted odds ratios (AORs) for every 1km increase in distance to maternity care on the use of skilled care at birth from adequately-adjusted analyses. Weights are for random-effects meta-analysis. PHC = primary health care; HC = health center; HF = health facility. *Unadjusted estimate used as adjusted estimate was unavailable.

Across all studies, women’s mean distances to their nearest HF of any level and specified HFs was 4km and 15km, respectively. Anastasi et al. 2015 [37] found no crude association between distance to the nearest HF with maternity care and use (S2 Table). Seven and 14 studies evaluating distance to the nearest HF and distance to one or more specified HF(s) reported effects controlled (or at least tested) for affordability, education and (perceived) need, respectively (Table 3). Among these 22 studies, four found distance to have no significant effect on use of skilled care at birth. The rest concluded that increased distance was at least marginally significantly associated with reduced use of skilled care at birth (p<0.1). From adequately-adjusted studies, meta-analysis indicated that every kilometre increase in distance to a source of maternity care was associated with a reduction in the odds of using skilled care at birth (pooled OR = 0.90, 95%CI = 0.85–0.94) (Fig 4). There was, however, evidence of high heterogeneity (I^2^ = 96%, p-value<0.001).

**Table 3:** Effect estimates of multivariate association adjusted for affordability, education and need or perceived need for skilled care for childbirth (study details available in S2 Table. Summary of 57 included studies).

Study	Settings	(Reference)	Multivariate association with using skilled care for childbirth (95% confidence interval if available)										
**Distance to the nearest facility**													
De Allegri et al. 2011	Rural Burkina Faso	*≤5km*		>5km	0.035**								
Johnson et al. 2015	Rural Ghana	*≤8km*		>8km	0.74 (0.62–0.88)**								
Lwelamira and Safari 2012	Rural Tanzania	*<5km*		5-10km	0.87 (0.73–1.04)	>10km	0.62 (0.47–0.81)*						
Nakua et al. 2015	Rural Ghana	*≤5km*		6-10km	0.32 (0.13–0.74)*	11-15km	0.40 (0.11–1.46)						
Mageda et la. 2015	Rural Tanzania	*<5km*		5-10km	0.43 (0.5–1.7)	≥10km	0.43 (0.3–0.8)**						
O’Meara et al. 2014	Rural Kenya (multi-site)												
	- Butula	Every one kilometre increment: 1.33 (1.00–1.69)*											
	- Bunyala	Every one kilometre increment: 0.80 (0.60–1.07)											
	- Teso North	Every one kilometre increment: 1.14 (0.79–1.64)											
	- Bungoma East	Every one kilometre increment: 1.18 (0.93–1.51)											
Ndao-Brunblay et al. 2014	Rural Tanzania	Every one kilometre increment: 0.89 (p-value = 0.085)											
**Distance to health facilities equipped to provide skilled care for childbirth**													
Mpembeni et al. 2007	Rural Tanzania	*0-5km*		6+km	0.25 (0.16–0.37)***								
De Allegri et al. 2015	Rural Burkina Faso	*≤6km*		7km	(0.01–0.30)*								
Moran et al. 2006	Rural Burkina Faso	*<22*.*8km*		≥22.8km	0.39 (0.202–0.759)*								
Mills et al. 2008	Urban and rural Ghana	*0-9km*		10-19km	0.54 (0.37–0.79)*	20+km	0.31 (0.23–0.43)*						
Magadi et al. 2000	Urban and rural Kenya	*<5km*		5-10km	0.47*	>10km	0.38*						
Gage 2007	Rural Mali	*<1km*		1-4km	0.526 (0.277–1.001)	5-9km	0.491 (0.277–0.871)*	10-14km	0.418 (0.212–0.825)*	15-29km	0.403 (0.209–0.779)***	30+km	0.623 (0.262–1.480)
Okafor 1991	Rural Nigeria	Every one kilometre increment: -0.097*											
Lohela et al. 2012	Rural Malawi	Every one^$^ kilometre increment: 0.90 (0.87–0.93)***											
Kruk et al. 2015	Rural Tanzania	Every one kilometre increment to dispensary (equipped to provide maternity care): 0.93 (0.84–1.04)											
		Every one kilometre increment to primary health clinics (equipped to provide maternity care): 1.07 (0.97–1.19)											
		Every one kilometre increment to hospital (higher-level and provide maternity care): 0.40 (0.26–0.63)***											
Hounton et al. 2008	Rural Kenya	Every one kilometre increment to health centre (lower-level and usually led by a nurse) for <7.5km: 0.77 (0.75–0.79)***											
		Every one kilometre increment to health centre (lower-level and usually led by a nurse) for ≥7.5km: 0.97 (0.95–0.98)***											
		Every one^$^ kilometre increment to hospital (higher-level and the main source of surgical care): 0.97 (0.97–0.99)***											
Joharifard et al. 2012	Rural Rwanda	Every one kilometre increment (up to 14): 0.909 (0.608–1.907) **INSIGNIFICANT**											
Kitui et al. 2013	Urban and rural Kenya	Adjusted effect of distance to the nearest HF offering maternity care is insignificant (results not presented in original study) **INSIGNIFICANT**											
Anyait et al. 2012	Mostly rural Uganda	Adjusted effect of distance to the nearest HF offering maternity care is insignificant (results not presented in original study) **INSIGNIFICANT**											
Nuwaha and Amooti-kaguna 1999	Mostly rural Uganda	Adjusted effect of distance to the nearest HF offering maternity care is insignificant (results not presented in original study) **INSIGNIFICANT**											
**Travel time**													
Wado et al. 2013^+^	Urban and rural Ethiopia	*<60min*		>60min	0.55 (0.34–0.89)*								
Hailu et al. 2014^+^	Urban and rural Ethiopia	*<60min*		≥60min	0.3 (0.11–0.87)*								
Gebru et al. 2014^+^	Ethiopia	*≤60min*		>60min	0.249 (0.143–0.434)**								
Abikar et al. 2013	Kenya	*<60min*		>60min	0.26 (0.08–0.81)*								
Van Eijk et al. 2006^+^	Rural Kenya	*<60min*		60min	0.58 (0.33–1.05)	>60min	0.36 (0.18–0.75)*						
Spangler and Bloom 2010^+^	Rural Tanzania	*<30min*		30-60min	0.45 (0.31–0.64)***	≥60min	0.26 (0.18–0.38)***						
Kawakatse et al. 2014^+^	Rural Kenya	*<20min*		21-40min	0.547 (0.536–0.558)	41-60min	0.533 (0.515–0.554)	>60min	0.403 (0.282–0.573)***				
Masters et al. 2013^++^	Rural Ghana	Every one hour increment: 0.801 (0.69–0.93)***											
Teferra et al. 2012^+^	Urban and rural Ethiopia	Adjusted effect of walking time is insignificant (results not presented in original study) **INSIGNIFICANT**											
Amano et al. 2012^+^	Urban and rural Ethiopia	Adjusted effect of walking time is insignificant (results not presented in original study) **INSIGNIFICANT**											
Nuwaha and Amooti-kaguna 1999^+^	Mostly rural Uganda	Adjusted effect of motorized travel time is insignificant (results not presented in original study) **INSIGNIFICANT**											

Reverse associations shown if original results were presented with greater distance or travel time as reference category or unskilled care for childbirth as the outcome of interest.

^$^ Association shown was converted from a 10-km increment by raising the original estimate to the power of 0.1.

^+^ Walking time;

^++^ Motorized travel time;

* p<0.05;

** p<0.01;

*** p<0.001

Hounton et al. 2008 [35], Mills et al. 2008 [30] and Kruk et al. 2015 [38] presented adjusted results and provided an opportunity to assess the effects of distance when different types of end location were compared. After controlling for distance to the nearest HF, increased distance to a higher-level HF remained strongly associated with a reduced likelihood of skilled care at birth in all three studies. However, meta-analysis of studies from rural areas of (Hounton et al. 2008 [35] and Kruk et al. 2015 [38]) showed no effect of increased distance (pooled aOR = 0.58, 95%CI = 0.31–1.09) and strong evidence of heterogeneity (I^2^ = 87.9%, p-value = 0.004) (Fig 4). Meta-analysis was also conducted on five estimates of distance to lower-level HFs (all set in rural areas). The result suggested a small but significant effect of distance (pooled aOR = 0.97, 95%CI = 0.96–0.99) with no evidence of heterogeneity (I^2^ = 41.7%, p-value = 0.143) (Fig 4).

### Measuring travel time

#### Quality assessment

The numbers of travel time measurements totalled 25, three of which were clearly defined with the start and end locations as well as the mode of transportation. Of these, one was well-defined as the walking time from women’s home to their nearest point of maternity care provision (Table 1).

#### Typology of travel time measurements

Table 2(b) shows the various travel time measurements identified. Two studies (Masters et al. 2013 [39] and Nesbitt et al. 2014 [32]) measured and estimated the travel time required to reach a HF by mapping population locations, health service locations, land-cover and detailed road networks in the study areas in GIS. Travel times from different locations where subpopulations resided to health services were then estimated on the basis of empirically derived driving/walking speed [40,41]. The rest of travel time measurements (n = 23) were self-reported data collected from surveys, where women were asked the time they would need to reach their nearest HF (n = 13) and a specified HF (n = 12). The start point of origin and the mode of transportation were unreported in 21 and 10 studies, respectively.

#### Effects of travel time

Fig 3(b) plots the 25 studies using the sample mean levels of skilled care utilization and mean travel times. No specific pattern emerged from these observations. The average walking time to the nearest HF was 68 minutes and time to reach all other specified facilities was 108 minutes.

Anastasi et al. 2015 [37] and Anyait et al. 2012 [42] found no crude relationship between travel time and utilization of skilled care at birth. Results of the 11 studies that reported the mode of transportation, a crucial element in understanding travel time, and were adequately-adjusted are shown in Table 3. Teferra et al. 2012 [43], Amano et al. 2012 [44], Nuwaha and Amooti-kaguna 1999 [45] found no significant adjusted association between walking time and utilization of skilled care at birth. Van Eijk et al. 2005 [46] and Kawakatse et al. 2014 [47] compared more than two categories of travel time and only found significant difference between the reference category and the furthest (longest time) category. The other six studies found significant reduced use of skilled care as travel time increased in every comparison made, including four studies (Wado et al. 2013 [48], Hailu et al. 2004 [49], Gebru et al. 2014 [50] and Abikar et al. 2013 [51]) that looked at times less than 60 minutes versus above, Spangler and Bloom 2010 [52] who compared <30 minutes, 30–60 minutes and ≥60 minutes, and lastly, Masters et al. 2013 [39] who considered motorized travel time as a continuous variable. Meta-analysis was not possible as only one study—the authors showed a reduced of 24% in odds of FBD in rural mothers per hour increase in motorized travel time [39].

## Discussion

### Summary of findings

In this systematic review we found evidence suggesting that increased distance and travel time to health facility were inversely correlated with the utilization of skilled care at childbirth in SSA. The details required for a meaningful and thorough understanding of the effect of spatial separation between women and health service were clearly provided by 12 of the 57 included studies and the effect of travel time by three studies. In addition, a few studies suggested that the negative effect of spatial separation waned for women who live very far from health services. For these populations who are perhaps “too” separated from healthcare provision, utilization of skilled birth attendance was generally very low and further increase in distance had no significant effect on skilled care utilisation.

### Limitations of existing evidence

These findings should be interpreted with certain limitations in mind. The 57 included studies were predominately retrospective and cross-sectional, and only one quarter of them primarily focused on physical accessibility. Many countries in SSA, including the whole sub-region of Central Africa, where within-country, urban-rural disparities in delivery care utilization have been noted [53,54], is entirely unrepresented. Substantial amount of included studies is prone to location and selection biases, considered non-home births (albeit some of these studies intended to examine the determinant of home deliveries) and childbirths in HFs of any level, or those attended by medical personnel of any qualifications as “skilled” care. However, these limitations likely result in an underestimate of the observed effect in the current review. Moreover, almost all studies relied on self-reported data for place of and attendant at childbirth, which could cause misclassification as respondents may not know or recall correctly the level of HF and the medical qualification of the birth attendant.

### Findings from systematic review

This review has identified two major ways to measure distance and travel time in the literature of maternity care utilization—self-reported data and GIS-based estimation using overlaid coordinates of the population and health services. In the former, the details required to thoroughly comprehend physical accessibility are often unreported. Kabakyenga and colleagues [55], for instance, did not report the mode of transportation although the questionnaire they had adopted included a question on how women went to the HF for labour [56]. Absence of such details could also be rooted in the lack of clarity in the survey instrument itself. An unclear question would lead to increased variability in the data as people interpret the question differently. Readers of these studies cannot reach a meaningful understanding without making strong assumptions, thus hindering the translation of quantitative findings to actionable information.

The more rigorous distance and travel time estimation techniques performed in a GIS are less prone to error induced from respondents not having a concrete idea of geographical space, and does not depend on people knowing where the nearest health facility/maternity care/hospital is located. However, its application would depend on the availability of geo-coded study sample and the local healthcare infrastructure—data often unavailable or costly to obtain in low-resource settings. To determine network distance would further require geospatial data of the road system. Nesbitt et al. 2014 [32] found no significant difference in the effects on facility-based delivery between straight line and road distances. Adaptation of similar investigation in a greater variety of contexts can strengthen such evidence base and justify the use of road distance in studies attempting to answer other related questions.

Researchers should avoid asking for self-reported distance and travel time as they can be difficult to conceptualize. Researchers of population-based study should capitalize on fieldworkers’ travels to collect location data, either as travel routes or fixed-point coordinates. For facility-based study, addresses should be collected and manually referenced on hardcopy maps or satellite imageries. It is surprising that the use of addresses and printed maps as means of assessing distance was scarce. In the current review, only the older studies quantified women’s physical accessibility to healthcare on printed maps [26,28]. Quality of the measurements made would depend on map resolution and the accuracy of manual referencing of addresses and locations (of women’s home/community and HFs). Nonetheless, this approach is particularly feasible for small-scale regional studies with engagement of the local communities. While detailed maps used to be difficult to obtain, the open-source community has started to address this challenge by making topographic information and crowdsourced map available [57]. Key features and landmarks are digitized to create shapefiles and vector layers of spatial objects that can then be imported and analysed in major analytical software packages broadly used in epidemiological studies (such as Stata, R, ArcGIS and QGIS). Multiple studies of maternity care determinants set in LMICs have already adopted this approach [33,58–61]. Prioritizing accurate, up to date, and reliable geospatial data availability has been highlighted in expert group meetings [62], but the compilation of geospatial data of health facility census and other ancillary data (e.g., road network) remains a challenge for local teams. Digitization of satellite imagery requires basic computational skills and is responsive to changes on the ground, offering an opportunity to fill current data gap.

On average, women from the identified studies live 15km and 108 minutes of walking time to a health facility likely to be equipped for skilled delivery. These levels are above the 5km threshold of what is considered walkable for a heavily pregnant woman [63], and the one-hour travel time to the nearest obstetric care recommended by the WHO [64]. Meta-analysis of adequately-adjusted results demonstrated that increased distance from maternity care provision deters use of maternity care for those with such intention and financial ability. Distance to a higher-level facility might have additional appeal to labouring women, despite accessibility to a nearby facility of lower-level (although unsupported by our meta-analysis possibly due to between-study heterogeneity and high within-study uncertainty in some instances). In addition to maternal and other individual and community factors, studies of bypassing front-line facilities for childbirth in LMICs have identified perceived higher quality of care, availability of drugs and medical equipment, and additional obstetric care functionality at higher-level HF as important determinants [65–71]. Investing in frontline facilities to ensure they have the appropriate equipment, drugs and medical personnel for their intended roles could increase met need for obstetric care within minimal travel time, especially in rural settings.

A few studies identified in the current review suggested distance and travel time to be influential only within a certain threshold, beyond which utilization is universally low and any extra spatial separation ceases to have an effect. Non-linearity should therefore be noted when analysing the effect of distance and travel time. We are unable to identify one universal critical threshold from available evidence. This is due to context-specificity, the many ways in which spatial separation has been captured, the different analytical approaches used in individual studies, as well as a lack of a reporting standard in the current literature.

Overcoming spatial separation requires bringing people to healthcare or healthcare to people [72]. From a policy-making perspective, modifiable health system factors such as, but not limited to, physical accessibility have contextual importance in prioritizing action and devising appropriate health system responses. Relevant strategies have been proposed in SSA and other LMICs [72–77]. Governments and their partners should prioritize the provision of better measurement to ensure countries have quality data to make informed decisions.

### Limitation of the systematic review

Physical accessibility, financial affordability and service acceptability form the basis of potential access [18,78]. To certain extent, affordability enables physical accessibility and service acceptability may change individuals’ perception of distance/travel time. We accounted for financial means directly, but not for service acceptability. This is because service acceptability is in part, among others, users’ social status and perception of need, which we have already addressed [79]. We omitted the grey literature and so might have missed some relevant studies. However, these are also the ones unlikely to (i) have novel approaches or tools not already identified and (ii) included a well-adjusted analysis. Only a small proportion of the included studies (25%) primarily addressed physical inaccessibility, which suggests possible publication bias from studies that intended to assess the influence of distance and travel time but did not find any significant association.

## Conclusion

Higher reporting standard of distance and travel time is needed to help understand and device appropriate strategies to overcome the persisting spatial separation between women and maternity care in SSA. Utilization is not possible without access and current evidence, while not without limitations, shows that suboptimal physical inaccessibility impedes use. In light of the global effort to reduce preventable maternal and newborn mortality and morbidity, researchers and policy makers should prioritize the provision of better measurement and information to ensure countries have quality data to make informed decisions on the spatial distribution of health facilities that provides physically accessible skilled delivery care to all women.

## Supporting information

S1 Table Complete search strategy. (DOCX)

S2 Table Summary of 57 included studies. (DOCX)

S3 Table PRISMA 2009 checklist. (DOC)

S1 Document Copyright permission from original copyright holder. (MSG)
