# Supplementary material for: A look back on how far to walk: Systematic review and meta-analysis of physical access to skilled care for childbirth in Sub-Saharan Africa
Source: PLoS One. 2017 Sep 14;12(9):e0184432. doi: 10.1371/journal.pone.0184432 (PMC5598961; doi:10.1371/journal.pone.0184432)
Supplement: S1 Table — (DOCX) [file pone.0184432.s001.docx]

## Search strategy

### Medline

*a. Sub-Saharan Africa*

| Africa, Western/ or Africa, Central/ or "Africa South of the Sahara"/ or Africa, Eastern/ or Africa, Southern/ | 16650 | OR 471187 |
| --- | --- | --- |
| Angola or Benin or Botswana or Burkina Faso or Burundi or Cameroon or Cape Verde or Central African Republic or CAR or Chad or Comoros or Congo or Cote d'Ivoire or cote dIvoire or Ivory Coast or DRC or Djibouti or Equatorial Guinea or Eritrea or Ethiopia or Gabon or Gambia or Ghana or Guinea or Guinea-Bissau or Kenya or Lesotho or Liberia or Madagascar or Malawi or Mali or Mauritania or Mauritius or Mozambique or Namibia or Niger or Nigeria or reunion or Rwanda or "Sao Tome and Principe" or Senegal or Seychelles or Sierra Leone or Somalia or South Africa or Sudan or Swaziland or Tanzania or Togo or Uganda or Western Sahara or Zambia or Zimbabwe or SSA or "sub-saharan Africa" | 366661 |  |
| Cross-culture comparison/ or developing countries/ or multicentre studies/ or | 111314 |  |
| multicountry or multi-country or multiple countries or multicentre or multicentre or multi-center or multi-centre |  |  |

b. *Geographic access*

| "Catchment area (health)"/ or Geographic information systems/ or Geographic mapping/ or Time factors/ or Travel/ or health service accessibility/ | 1093409 | OR 2035284 |
| --- | --- | --- |
| Geospatial or spatial or gis or "geographic information system" or "geographic information systems" or distance* or travel* or transport* | 918903 |  |
| time* | 69453 |  |
| adj5 |  |  |
| (birth* or childbirth? or deliver* or labo?r or parturition? or obstetric* or gyn?ecology or facilit* or hospital* or institut* or clinic? or center? or centre? or department? or unit? or ward?) |  |  |
| (km? or m? or kilometer? or meter? or mile?) | 58058 |  |
| adj2 |  |  |
| (at least or more or less or within or from or to or away or walk or drive or ride or bike or cycle or commut*) |  |  |
| (physical or geograph*) | 824 |  |
| adj1 |  |  |
| (inaccess* or access*) |  |  |

c. *Skilled care at birth*

| fbd or sba | 2600 |  | | OR 237791 |
| --- | --- | --- | --- | --- |
| (birth* or childbirth? or deliver* or labo?r or parturition?) | 41330 |  |  |  |
| adj5 |  |  |  |  |
| (facilit* or non-facilit* or nonfacilit* or hospital* or institut* or non-institut* or noninstitut* or clinic? or center? or centre? or department? or unit? or ward? or place or home* or domicile* or village* or domestic or community or assist* or attend) |  |  |  |  |
| (village or tradition* or skill* or train*) | 16173 |  |  |  |
| adj1 |  |  |  |  |
| (attend* or birth attend* or health or assistant* or care or manpower or delivery or staff or midwif* or professio*) |  |  |  |  |
| Birthing centers/ or Delivery rooms/ or Delivery, obstetric/ or Home childbirth/ | 27142 |  |  |  |
| Birth* or Childbirth? or Deliver* or Labo?r or Parturition or Pregnan* or | 1434254 |  | AND 176186 |  |
| Obstetrics/ or Parturition/ or Pregnancy/ |  |  |  |  |
| Physicians/ or doctor* or physician* or | 783285 |  |  |  |
| Midwifery/ or midwi* or nurses/ or nurse* or obstetrical nursing/ or |  | OR |  |  |
| Professional practice/ or Health personnel/ or ((clinical or health of medical) adj1 (officer* or auxiliary*)) |  |  |  |  |
| "Delivery of Health Care"/ or "Obstetrics and Gynecology Department, Hospital"/ or Health Behavior/ or Health facilities/ or Health Facility Closure/ or Health Personnel/ or Health Services/ or Healthcare Disparities/ or Maternal Health Services/ or Maternal-Child Health Centers/ or Universal Coverage/ | 190269 |  |  |  |
| ((health* or medical) adj3 (utiliz* or utilis* or use* or uptake* or access*)) | 141332 |  |  |  |

((a + b + c) OR (b + c [review])) limited to year > 1986

### Africa Wide Information

b. *Geographic access*

| Geospatial or spatial or travel or gis or "geographic information system" or "geographic information systems" or distance* or travel* or transport* | 136078 | OR  141818 |
| --- | --- | --- |
| (time*) | 3376 |  |
| W5 |  |  |
| (birth* or childbirth? or deliver* or labo?r or parturition? or obstetric* or gyn?ecology or facilit* or hospital* or institut* or clinic? or center? or centre? or department? or unit? or ward?) |  |  |
| (km? or m? or kilometer? or meter? or mile?)  W2  (at least or more or less or within or from or to or away or walk or travel or drive or ride or bike or cycle or commut*) | 2808 |  |
| (physical or geograph*) | 271 |  |
| W1 |  |  |
| (inaccess* or access*) |  |  |

c. *Skilled care at birth*

| fbd or sba | 3482 |  |  | OR  354294 |
| --- | --- | --- | --- | --- |
| (birth* or childbirth? or deliver* or labo?r or parturition?) | 63404 |  |  |  |
| adj5 |  |  |  |  |
| (facilit* or non-facilit* or nonfacilit* or hospital* or institut* or non-institut* or noninstitut* or clinic? or center? or centre? or department? or unit? or ward? or place or home* or domicile* or village* or domestic or community or assist* or attend) |  |  |  |  |
| (village or tradition* or skill* or train*) | 32082 |  |  |  |
| adj1 |  |  |  |  |
| (attend* or birth attend* or health or assistant* or care or manpower or delivery or staff or midwif* or professio*) |  |  |  |  |
| Birth* or Childbirth? or Deliver* or Labo?r or Parturition or Pregnan* or | 1721875 |  | AND  285872 |  |
| Birth/or Childbirth/ or Obstetrics/ or Parturition/ or Pregnancy/ |  |  |  |  |
| Physician/ or doctor* or physician* or  Midwife/ or Nurse/ or Nurse midwife/ or Nurse midwifery/ or midwi* or nurse* or  Health auxiliary/ or Health care manpower/ or Health care personnel/ or Medical personnel/ or Professional practice/ or  ((clinical or health of medical) adj1 (officer* or auxiliary*)) | 1100487 | OR |  |  |
| Health care delivery/ or Health care facility/ or Health care utilization/ or Health care/ or Health center/ or Health service/ or  Hospital service/ or Hospital utilization/ or Medical service/ or Public health service/ or  Maternal care/ or Maternal treatment/ or Maternity ward/ | 538964 |  |  |  |
| ((health* or medical) adj3 (utiliz* or utilis* or use* or uptake* or access*)) | 197683 |  |  |  |

b + c

### Global health

a. *Sub-Saharan Africa*

| Africa, Western/ or Africa, Central/ or "Africa South of the Sahara"/ or Africa, Eastern/ or Africa, Southern/ | 165042 | OR  803358 |
| --- | --- | --- |
| Angola or Benin or Botswana or Burkina Faso or Burundi or Cameroon or Cape Verde or Central African Republic or CAR or Chad or Comoros or Congo or Cote d'Ivoire or cote dIvoire or Ivory Coast or DRC or Djibouti or Equatorial Guinea or Eritrea or Ethiopia or Gabon or Gambia or Ghana or Guinea or Guinea-Bissau or Kenya or Lesotho or Liberia or Madagascar or Malawi or Mali or Mauritania or Mauritius or Mozambique or Namibia or Niger or Nigeria or reunion or Rwanda or "Sao Tome and Principe" or Senegal or Seychelles or Sierra Leone or Somalia or South Africa or Sudan or Swaziland or Tanzania or Togo or Uganda or Western Sahara or Zambia or Zimbabwe or SSA or "sub-saharan Africa" | 221215 |  |
| Developing countries/ or least developed countries/ or international comparisons/ or | 787861 |  |
| multicountry or multi-country or multiple countries or multicentre or multicentre or multi-center or multi-centre |  |  |

b. *Geographic access*

| Access/ or Distance travelled/ or Geographical information systems/ or Mapping/ or Travel/ | 9712 | OR  130848 |
| --- | --- | --- |
| Geospatial or spatial or travel or gis or "geographic information system" or "geographic information systems" or distance* or travel* or transport* | 101663 |  |
| (time*) | 14216 |  |
| adj5 |  |  |
| (birth* or childbirth? or deliver* or labo?r or parturition? or obstetric* or gyn?ecology or facilit* or hospital* or institut* or clinic? or center? or centre? or department? or unit? or ward?) |  |  |
| (km? or m? or kilometer? or meter? or mile?) | 13051 |  |
| adj2 |  |  |
| (at least or more or less or within or from or to or away or walk or travel or drive or ride or bike or cycle or commut*) |  |  |
| (physical or geograph*) | 460 |  |
| adj1 |  |  |
| (inaccess* or access*) |  |  |

c. *Skilled care at birth*

| fbd or sba | 497 |  |  | OR  43473 |
| --- | --- | --- | --- | --- |
| (birth* or childbirth? or deliver* or labo?r or parturition?) | 13049 |  |  |  |
| adj5 |  |  |  |  |
| (facilit* or non-facilit* or nonfacilit* or hospital* or institut* or non-institut* or noninstitut* or clinic? or center? or centre? or department? or unit? or ward? or place or home* or domicile* or village* or domestic or community or assist* or attend) |  |  |  |  |
| (village or tradition* or skill* or train*) | 6248 |  |  |  |
| adj1 |  |  |  |  |
| (attend* or birth attend* or health or assistant* or care or manpower or delivery or staff or midwif* or professio*) |  |  |  |  |
| Birth* or Childbirth? or Deliver* or Labo?r or Parturition or Pregnan* or | 212185 |  | AND  30082 |  |
| Birth/or Childbirth/ or Obstetrics/ or Parturition/ or Pregnancy/ |  |  |  |  |
| physicians/ or doctor* or physician* or | 86456 | OR |  |  |
| midwives/ or midwi* or nurses/ or nurse* or |  |  |  |  |
| medical auxiliaries/ or health care workers/ or |  |  |  |  |
| ((clinical or health of medical) adj1 (officer* or auxiliary*)) |  |  |  |  |
| Institutions/ or Health services/ or Hospitals/ or Health centres/ or Maternity service/ or Health care utilization/ | 84166 |  |  |  |
| ((health* or medical) adj3 (utiliz* or utilis* or use* or uptake* or access*)) | 34561 |  |  |  |

d. *Review*

| review or literature reviews/ or systematic reviews/ or reviews/ | 251068 |
| --- | --- |

((a + b + c) OR (b + c + d)) limited to year > 1986

### Popline

a. *Sub-Saharan Africa*

| Angola OR Benin OR Botswana OR Burkina Faso OR Burundi OR Cameroon OR Cape Verde OR Central African Republic OR CAR OR Chad OR Comoros OR Congo OR Cote d'Ivoire OR IvORy Coast OR DRC OR Djibouti OR Equatorial Guinea OR Eritrea OR Ethiopia OR Gabon OR Gambia OR Ghana OR Guinea OR Guinea-Bissau OR Kenya OR Lesotho OR Liberia OR Madagascar OR Malawi OR Mali OR Mauritania OR Mauritius OR Mozambique OR Namibia OR Niger OR Nigeria OR reunion OR Rwanda OR "Sao Tome and Principe" OR Senegal OR Seychelles OR Sierra Leone OR Somalia OR South Africa OR Sudan OR Swaziland OR Tanzania OR Togo OR Uganda OR Western Sahara OR Zambia OR Zimbabwe OR SSA OR "sub-saharan Africa" | OR |
| --- | --- |
| multicountry OR multi-country OR multiple countries OR multicentre OR multicentre OR multi-center OR multi-centre |  |
| LITERATURE REVIEW |  |
|  |  |

b. *Geographic access*

| Geospatial OR spatial OR travel OR gis OR "geographic information system" OR "geographic information systems" OR distance* OR travel* OR transport* or  "geographic access" OR "geographic accessibility" OR "geographic inaccess" OR "geographic inaccessibility" or  "geographical access" OR "geographical accessibility" OR "geographical inaccess" OR "geographical inaccessibility" or  "physical access" OR "physical accessibility" OR "physical inaccess" OR "physical inaccessibility" | OR |
| --- | --- |
| DISTANCE OR GEOGRAPHIC FACTORS OR TRANSPORTATION OR COMMUTING OR PROGRAM ACCESSIBILITY |  |

c. *Skilled care at birth (i)*

| "facility-based delivery" OR "facility based delivery" OR "facility-based birth" OR "facility based birth" OR "institutional delivery" OR "institutional birth" OR  "skilled birth" OR "skilled attendant" OR "skilled attendants" OR  "skilled assistant" OR "skilled assistants" OR "skilled assistance" OR  "traditional birth" OR "traditional attendant" OR "traditional attendants" OR  "traditional assistant" OR "traditional assistants" OR "traditional assistance" OR  SBA OR FBD OR homebirth | OR |
| --- | --- |
| TRADITIONAL BIRTH ATTENDANTS |  |

d. *Skilled care at birth (ii)*

| labour* OR labor* OR birth* OR childbirth* OR intrapartum OR intra-partum OR parturition* | OR |
| --- | --- |
| UTILIZATION OF HEALTH CARE |  |

e. *Skilled care at birth (iii)*

| labour* OR labor* OR birth* OR childbirth* OR intrapartum OR intra-partum OR parturition* | OR |
| --- | --- |
| DELIVERY OF HEALTH CARE |  |

(a + b + c ) OR (a + b + d) OR (a + b + e)

### EMBASE

a. *Sub-Saharan Africa*

| Africa, Western/ or Africa, Central/ or "Africa South of the Sahara"/ or Africa, Eastern/ or Africa, Southern/ | 12547 | OR  548123 |
| --- | --- | --- |
| "Africa south of the Sahara"/ or “Central Africa”/ or “North Africa”/ | 428257 |  |
| Angola or Benin or Botswana or Burkina Faso or Burundi or Cameroon or Cape Verde or Central African Republic or CAR or Chad or Comoros or Congo or Cote d'Ivoire or Ivory Coast or DRC or Djibouti or Equatorial Guinea or Eritrea or Ethiopia or Gabon or Gambia or Ghana or Guinea or Guinea-Bissau or Kenya or Lesotho or Liberia or Madagascar or Malawi or Mali or Mauritania or Mauritius or Mozambique or Namibia or Niger or Nigeria or reunion or Rwanda or "Sao Tome and Principe" or Senegal or Seychelles or Sierra Leone or Somalia or South Africa or Sudan or Swaziland or Tanzania or Togo or Uganda or Western Sahara or Zambia or Zimbabwe or SSA or "sub-saharan Africa" | 129953 |  |
| Developing countries/ or “multicentre study (topic)” or  multicountry or multi-country or multiple countries or multicentre or multicentre or multi-center or multi-centre |  |  |

b. *Geographic access*

| "traffic and transport"/ or Geographic information system/ or geographic mapping/ or geography/ or spatial analysis/ or travel/ | 86661 | OR  1440775 |
| --- | --- | --- |
| Geospatial or spatial or travel or gis or "geographic information system" or "geographic information systems" or distance* or travel* or transport* | 124408 |  |
| (time*) | 108745 |  |
| adj5 |  |  |
| (birth* or childbirth? or deliver* or labo?r or parturition? or obstetric* or gyn?ecology or facilit* or hospital* or institut* or clinic? or center? or centre? or department? or unit? or ward?) |  |  |
| (km? or m? or kilometer? or meter? or mile?)  Adj2  (at least or more or less or within or from or to or away or walk or travel or drive or ride or bike or cycle or commut*) | 77517 |  |
| (physical or geograph*) | 1079 |  |
| adj1 |  |  |
| (inaccess* or access*) |  |  |

c. *Skilled care at birth*

| fbd or sba | 3482 |  |  | OR  354294 |
| --- | --- | --- | --- | --- |
| (birth* or childbirth? or deliver* or labo?r or parturition?) | 63404 |  |  |  |
| adj5 |  |  |  |  |
| (facilit* or non-facilit* or nonfacilit* or hospital* or institut* or non-institut* or noninstitut* or clinic? or center? or centre? or department? or unit? or ward? or place or home* or domicile* or village* or domestic or community or assist* or attend) |  |  |  |  |
| (village or tradition* or skill* or train*) | 32082 |  |  |  |
| adj1 |  |  |  |  |
| (attend* or birth attend* or health or assistant* or care or manpower or delivery or staff or midwif* or professio*) |  |  |  |  |
| Birth* or Childbirth? or Deliver* or Labo?r or Parturition or Pregnan* or | 1721875 |  | AND  285872 |  |
| Birth/or Childbirth/ or Obstetrics/ or Parturition/ or Pregnancy/ |  |  |  |  |
| Physician/ or doctor* or physician* or  Midwife/ or Nurse/ or Nurse midwife/ or Nurse midwifery/ or midwi* or nurse* or  Health auxiliary/ or Health care manpower/ or Health care personnel/ or Medical personnel/ or Professional practice/ or  ((clinical or health of medical) adj1 (officer* or auxiliary*)) | 1100487 | OR |  |  |
| Health care delivery/ or Health care facility/ or Health care utilization/ or Health care/ or Health center/ or Health service/ or  Hospital service/ or Hospital utilization/ or Medical service/ or Public health service/ or  Maternal care/ or Maternal treatment/ or Maternity ward/ | 538964 |  |  |  |
| ((health* or medical) adj3 (utiliz* or utilis* or use* or uptake* or access*)) | 197683 |  |  |  |

((a + b + c) OR (b + c [review])) limited to year > 1986
