# Supplementary material for: A look back on how far to walk: Systematic review and meta-analysis of physical access to skilled care for childbirth in Sub-Saharan Africa
Source: PLoS One. 2017 Sep 14;12(9):e0184432. doi: 10.1371/journal.pone.0184432 (PMC5598961; doi:10.1371/journal.pone.0184432)
Supplement: S2 Table — (DOCX) [file pone.0184432.s002.docx]

***Studies included in Table 3***

**(i) Distance to any nearest health facility**

| **Citation**  **Country**  **Region**  **(Settings)** | **Study sample** | | | **Distance/travel time (exposure) measurement** | | | | **Study outcome** | | | **Results** | | | |
| --- | --- | --- | --- | --- | --- | --- | --- | --- | --- | --- | --- | --- | --- | --- |
|  | **Sampling design,**  **health facility data (where applicable) and sample size** | **Potential bias** | | **Distance**  **vs.**  **time** | **Line/**  **Transport**  **type** | **Start-end** | **SR vs.**  **Est.^1^** | **Birth location/ attendant** | **Skilled**  **care** | **Unskilled**  **care** | **Crude/Adjusted analysis;**  **summary of key results** | **Adjustment** | | |
|  |  | **Location** | **Sample**  **selection** |  |  |  |  |  |  |  |  | **Affordability** | **Education** | **(Perceived) need** |
| De Allegri et al. 2011^1^  Burkina Faso  Nouna  (Rural) | Multi-stage cluster random sample; first, clusters were defined according to the catchment area of each first-line HF. Second, one village where the HF was located, and another village randomly drawn from the list of all the villages in the HF catchment area were selected. In the third stage, 20 households in each village were randomly selected.  N=435 women pregnant within a one-year recall period | ✓ | ✕ | Distance | Unclear | Unclear  TO  Any nearest HF | SR | Location | HF (first-line HF or hospital) | Others | Adjusted; negative;  living <5km from a HF was associated with delivering in a HF – AOR=28.42, p-value<0.001 | Household asset index quintile | Maternal and paternal literacy | Parity |
| Johnson et al. 2015^2^  Ghana  Ghana  (Rural) | Multi-stage cluster random sample from the 2003 and 2008 Ghana DHSs, rural clusters only. The births recorded in the two surveys cover the periods 1999-2003 and 2004-2008.  Geo-referenced database of HFs, Community-based Health Planning and Services (CHPS) compounds and digitised topographic databased of national road network.  N=4349 births from the two DHSs within each of the two five-year recall periods | ✕ | ✕ | Distance | Road | Unclear  TO  Any nearest HF for CHPS compound (where provision of skilled delivery care is not stated as a core activity) | Est. | Attendant | People with midwifery skills (e.g. doctors, midwives, nurses) | Others | Adjusted; negative;  access to HF and CHPS (<8km) have a significant impact on the uptake of skilled birth care: the odds of skilled birth care increased by 35% (p<0.01) for those with access to HF, the odds of skilled care increased by 51% (p<0.05) for those with access to HF and CHPS. But living close to CHPS only showed no significant effect (p>0.05) | Household wealth status | Maternal education | Parity |
| Lwelamira and Safari 2012^3^  Tanzania  Bahi  (Rural) | Random selection of households from all households in the study area. Obstetric history of all women from sampled households were obtained.  N=984 women given birth within the two-year recall period | ✕ | ✕ | Distance | Unclear | Unclear  TO  Any nearest HF | SR | Location | HF | Others | Adjusted; negative;  Women >10km were 38% less likely to deliver in a HF compared to those <5km (AOR=0.62, 95%CI=0.47-0.81). But odds of facility delivery for those living 5-10km to the nearest HF were not significantly different from women <5km. | Annual household income | Maternal education | Perceived quality of maternity services |
| Nakua et al. 2015^4^  Ghana  Amansie West  (Rural) | The eligible study sample was respondents attending post-partum care identified by a sample of local health officials, and may not include mothers who were not engaged with the health system.  N=400 women given birth within the one-year recall period | ✕ | ✓ | Distance | Unclear | Unclear  TO  Any nearest HF | SR | Attendant | Persons with midwifery skills  (doctor, nurse, midwives and health officer) | Others | Adjusted; negative;  compared to <5km, living 6-10km to the nearest HF was associated with reduced odds of delivering in a HF – AOR=0.32 (95%CI=0.13,0.74). Living 11-15km to the nearest HF, however, did not show any significant effect. | Average household income per day | Maternal education | Ever used unskilled care, knowledge about benefits of skilled delivery |
| Mageda et al. 2015^5^  Tanzania  Biharamula  (Unclear) | Multi-stage cluster random sample  N=598 women given birth within the one-year recall period | ✕ | ✕ | Distance  _________  Time | Unclear  _________  Walking | Unclear  TO  Any nearest HF | SR | Location | HF | Others | Adjusted; negative;  women living <5km were more likely to have an institutional delivery than those living >10km, AOR=2. (95%CI=2.3-3.9). Odds for those living 5-10km were not significantly different from those living >10km.  ______________________________  No result was presented. | Regularity of maternal and paternal sources of income | Maternal and paternal education | Parity |
| O’Meara et al. 2014^6^  Kenya  Bunyala  (Rural) | Multi-stage cluster random sample data were collected from four districts within the Academic Model Providing Access to Healthcare (AMPATH) Primary  Health Care catchment area and may not represent household located outside of any catchment area.  GPS coordinates were captured for every household and HF.  N=1987 women who delivered in the last five years | ✓ | ✕ | Distance | Straight | Home  TO  1 Any nearest HF  2 Hospital | Est. | Location | Hospital/  Nursinghome, Health centre/  Dispensary, and private clinics | Home | (Any nearest HF) Adjusted; mixed;  effect of distance differed across the four districts – in Bunyala, Teso North and Bungamo East, distance was insignidicant but in Butula, distance was associated with delivery in a facility, AOR=1.33, 95%CI=1.00-1.69.  (Hospital) Crude; mixed;  significant only in Bunyala – COR=0.79, 95%CI=0.74-0.87. | Household SES index quartiles | Maternal education | Parity |
| Ndao-Brumblay et al. 2013^7^  Tanzania  Kasulu  (Rural) | Multi-stage cluster random sample; individual responses for distance to any nearest HF were aggregated at the village level and corresponding village estimates were assigned to individual respondents.  N=1183 women given birth within a five-year recall period | ✕ | ✕ | Distance | Unclear | Village  TO  Any nearest HF | SR | Location | Any government, mission or private HF | Friend’s home or own home | Adjusted; marginally negative;  distance from had a marginal negative association with institutionalized delivery (AOR=0.89, p=0.085). | Household asset index quintile | Maternal education | Parity |

**(ii) Distance to one or more specified facilities**

| **Citation**  **Country**  **Region**  **(Settings)** | **Study sample** | | | **Distance/travel time (exposure) measurement** | | | | **Study outcome** | | | **Results** | | | |
| --- | --- | --- | --- | --- | --- | --- | --- | --- | --- | --- | --- | --- | --- | --- |
|  | **Sampling design,**  **health facility data (where applicable) and sample size** | **Potential bias** | | **Distance**  **vs.**  **time** | **Line/**  **Transport**  **type** | **Start-end** | **SR vs.**  **Est.^1^** | **Birth location/ attendant** | **Skilled**  **care** | **Unskilled**  **care** | **Crude/Adjusted analysis;**  **summary of key results** | **Adjustment** | | |
|  |  | **Location** | **Selection** |  |  |  |  |  |  |  |  | **Affordability** | **Education** | **(Perceived) need** |
| Mpembeni et al. 2007^8^  Tanzania  Mtwara  (Rural) | A random sample HF was first selected. For each of the selected HF, one village in its catchment area was selected randomly. In the selected village, a house to house survey was conducted and all women who had given birth within the previous one year were interviewed.  N=974 women given birth within the one-year recall period | ✓ | ✕ | Distance | Unclear | Unclear  TO  Nearest HF with maternity care | SR | Attendant | Doctor, nurse, midwife, MCHA  (TBA unclear) | Untrained relatives or friends  (TBA unclear) | Adjusted; negative;  distance to the HF providing maternity care (AOR=4.09, 95%CI=2.72–6.16)  were significant associated with use of skilled care at birth. | Household asset index quintile | Maternal education | Advised where to deliver during ANC and knowledge of pregnancy danger signs |
| De Allegri et al. 2015^9^  Burkina Faso  Nouna  (Rural) | Multi-stage cluster random sample with women who resided within the catchment areas of front-line health facilities only.  N=420 women given birth within the one-year recall period | ✓ | ✕ | Distance | Unclear | Village  TO  Specified front-line HF equipped as BEmOC | Unclear | Location | Others | Home | Adjusted; negative;  there were significant association between home delivery and greater distance to preassigned front-line HF (AOR=19.33, 95%CI=3.37-110.88). | Household asset index quintile | Maternal and household head’s literacy | History of miscarriage |
| Moran et al. 2006^10^  Burkina Faso  Koupela  (Rural) | The study area comprised 145 villages in 13 health facility catchment areas. Within each catchment area, villages were stratified as further than or within the average distance to the HF. Villages from stratum were randomly selected, and women were then random selected from chosen villages.  N=180 women given birth within the one-year recall period | ✓ | ✕ | Distance | Unclear | Unclear  TO  Preassigned health centre | Unclear but likely to be measured | Attendant | Doctor, nurse, midwife or auxiliary nurse | Others | Adjusted; negative;  women living further away were 61% (95%CI=24.4-79.8) less likely to use a skilled provide at delivery. | Plan for saving money for emergency | Maternal education | Parity |
| Mills et al. 2008^11^  Ghana  Kassena-Nankana  (Urban and rural) | All mothers with recorded birth in the local Health and Demographic Surveillance Site.  The NDSS has a geographical information system with a geo-reference of roads, HFs rivers, and households/compounds. | ✕ | ✓ | Distance | Road | Cluster  TO  1 Nearest HF  2 District hospital | Est. | Attendant | Health professional (doctor, midwife or nurse) | Others | Adjusted; negative;  distance to the district hospital showed significant association with use of health professionals at last delivery – AOR for >20km compared to <10 km was 0.31 (95%CI=0.33,0.43) and AOR for 10-19km compared to <10km was 0.54 (95%CI=0.34-0.79).  Effect of distance to nearest HF was insignificant. | Household asset index quintile | Maternal education | Parity |
| Magadi et al. 2000^12^  Kenya  Kenya  (Urban and rural) | Multi-stage cluster random sample from the 1993 Kenya DHS  N=5,290 births occurred within the five-year recall period | ✕ | ✕ | Distance  _________  Time | Unclear  _________  Unclear | Unclear  TO  Nearest HF with maternity care | SR | Location | Others | Home | Adjusted; negative;  for births occurring 5-10km or >10km away, the average odds of home births are more than double, compared to births occurring <5km from maternity care.  ______________________________  Adjusted; negative;  the average odds of home births >2h from maternity care are almost double the odds of births <1h from a HF offering maternity care. | Household asset index tercile | Maternal education | Birth Order |
| Gage 2007^13^  Mali  Mali  (Rural) | Multi-stage cluster random sample from the 2001 Mali DHS, including a community questionnaire that collected data from key informants on the socioeconomic, and health and other infrastructure of enumeration areas selected for interview.  N=6,178 most recent births within the five-year recall period | ✕ | ✕ | Distance | Unclear | Unclear  TO  Nearest HF with maternity care | SR (by community informants) | Attendant  _________  Location | Trained health worker  (doctor, nurse, auxiliary nurse or midwife)  _________  HF | Others  _________  Others | Adjusted; negative;  Distance had strong effects on the odds of both facility-based delivery and delivering with a trained health worker. | Household asset index | Maternal education | Mother told about pregnancy complications |
| Okafor 1991^14^  Nigeria  Udi  (Rural) | One-stage sample of town in part based on interviewers’ convenience and all women residing were interviewed.  N=498 women given birth within the two-year recall period | ✓ | ✕ | Distance | Unclear | Town  TO  Nearest HF with maternity care | SR | Both | In a HF with at least a midwife or a trained and state licenced auxiliary/  TBA | Others | Adjusted; negative; effect of distance was significant at p<0.05 level. | Husband’s occupation | Maternal education | Parity |
| Lohela et al. 2012^15^  Malawi  Malawi  (Rural)  Note: We also included Gabrysch et al. 2011^16^ which covered the analysis for Zambia in Lohela et al. 2012. | Multi-stage cluster random sample from the 2004 Malawi DHS, rural clusters only. Births that occurred before the mothers moving to the current location were excluded.  Facility data on all public and semi-public and major private HFs were obtained from national Health Facility Censuses conducted in Malawi in 2002.  N=8842 children born within the five-year recall period | ✕ | ✕ | Distance | Straight | Cluster  TO  Nearest HF with maternity care | Est. | location | HF | Others | Adjusted; negative;  the odds of facility delivery decreased by  65% for every 10 km increase in distance to the closest facility (AOR=0.35, p=0.001). | Household asset index quintile | Paternal education | Women’s modern attitudes (in cluster) |
| Kruk et al. 2015^17^  Tanzania  Pwani  (Rural) | The study population is women with deliveries in the past year who live in catchment areas of 24 study health facilities: government primary care clinics with at least one medically trained staff member who were trained in basic obstetric care and were actively providing delivery services.  Enumerators collected locations of all HFs in the study area and sub-village centres using GPS receivers.  N=3,019 women given birth between six weeks and one year before interview | ✓ | ✕ | Distance | Straight | Village  TO  1 Primary clinic  2 Health centre  3 Hospital | Est. | Location | HF | Home (own or that of someone else) | Adjusted; negative;  women’s distance from the nearest hospital was strongly associated with an increased likelihood of home delivery (AOR=2.49, 95%CI=1.60,3.88). Distance from the nearest health center and dispensary were not associated with likelihood of home delivery. | Household asset index quintile | Maternal education | Primipara |
| Hounton et al. 2008^18^  Burkina Faso  Ouargaye  (Rural) | A census was conducted to cover the total population in the study area.  All 43 health facilities in the two districts were surveyed, each of which typically led by a nurse and maternity care typically provided by an auxiliary midwife, except for in remote centres where TBA are the main givers of maternity care.  N=81,539 women given birth within the five-year recall period. | ✕ | ✕ | Distance | Unclear | Unclear  TO  1 Preassigned health centre (typically led by a nurse)  2 Preassigned hospital | Unclear | Location | Health centre or hospital | Others | Adjusted; negative;  the effect of distance to the health centre was very pronounced up to about 7 km from the health centre (AOR 0.77⁄km, 95%CI=0.75-0.79), levelling off beyond that (AOR 0.97⁄km, 95%CI=0.95-0.98). Distance to the district hospital remained an important predictor of institutional birth – AOR 0.83/10km, 95%CI=0.77-0.91) | Household asset index quintile | Maternal education | Parity |
| Joharifard et al. 2012^19^  Rwanda  Bugesera  (Mostly rural) | From each selected villages, 40 women were to be interviewed. Enumerators stood from the main road at the edge of each village, approached the closest households consecutively until they had either approached all households or interviewed 40 women.  N=959 women given birth within the three-year recall period | ✓ | ✕ | Distance | Road | Village  TO  1 Each village’s designed health centre (staffed exclusively by nurse)  2 Nyamata District Hospital  (result not presented) | Est. | Location | Health centre, hospital | Others | Adjusted; negative;  Greater distance between the respondents’’ village and her designated HC was negatively associated with facility delivery – AOR=0.909 (95%CI=0.846-0.976) per additional kilometre. | Covered by health insurance | Maternal education | Next-to-last delivery at HF, past intra/post-  partum problems |
| Kitul et al. 2013^20^  Kenya  Kenya  (Urban and rural) | Multi-stage cluster random sample of the 2008-2009 Kenya DHS; HF data from the 2008 Kenya Health Facility Database  N=5857 live births within a five-year recall period | ✕ | ✕ | Distance | Straight | Cluster  TO  Nearest HF with maternity care | Est. | Location | Any HF | Home or on the way | Adjusted; insignificant;  results not presented. | Household asset index quintile | Maternal education | Parity |
| Anyait et al. 2012^21^  Uganda  Busia  (Mostly rural) | The study population was restricted to women residing within 5km of a HF providing delivery services.  N=500 women given birth within a twp-year recall period | ✓ | ✕ | Distance  _________  Time | Unclear  **_________**  Unclear | Unclear  TO  Nearest HF with maternity care | SR | Location | Public and private HF | Others | Crude; negative;  living <3km of a HF offering maternity care increased the likelihood of HF delivery (COR=1.9, 95%CI=1.2-3.1)  Adjusted; insignificant;  result not presented  ______________________________  Crude; insignificant;  Time taken to reach maternity HF did not influence the place of delivery (COR=0.8, 95%CI=0.5-1.3). |  |  |  |
| Nuwaha and Amooti-kaguna 1999^22^  Uganda  Rakai  (Mostly rural) | Villages were selected proportional to population size, then standing in the centre of each village.  In addition, about 80% of the study population lived <5km from a HF  N=211 women given birth within the one-year recall period | ✕ | ✕ | Distance | Unclear | Unclear  TO  1 Nearest HF with maternity care  2 Nearest HF offering caesarean section (CS) | SR | Location | TBA’s place or HF | Home | Crude; negative;  39% of mothers <5km to a maternity centre delivered at home, compared with 66% who lived >5km (COR=0.35, 95%CI=0.17-0.71).  26% of the mothers who were <5km to a health unit that could do CS delivered at home, compared to 65% who were >5km (COR=0.21, 95%CI=0.11-0.40)  Adjusted; insignificant,  result not presented. |  |  |  |

**(iii) Walking or motorized travel time to any end location**

| **Citation**  **Country**  **Region**  **(Settings)** | **Study sample** | | | **Distance/travel time (exposure) measurement** | | | | **Study outcome** | | | **Results** | | | |
| --- | --- | --- | --- | --- | --- | --- | --- | --- | --- | --- | --- | --- | --- | --- |
|  | **Sampling design,**  **health facility data (where applicable) and sample size** | **Potential bias** | | **Distance**  **vs.**  **time** | **Line/**  **Transport**  **type** | **Start-end** | **SR vs.**  **Est.^1^** | **Birth location/ attendant** | **Skilled**  **care** | **Unskilled**  **care** | **Crude/Adjusted analysis;**  **summary of key results** | **Adjustment** | | |
|  |  | **Location** | **Selection** |  |  |  |  |  |  |  |  | **Affordability** | **Education** | **(Perceived) need** |
| Wado et al. 2013^23^  Ethiopia  Gilgel Gibe  (Urban and rural) | Multi-stage cluster random sample of women taken from mothers with recorded birth in the Gilgel Gibe Health and Demographic Surveillance Site.  N=1,456 women given birth within the two-year recall period | ✕ | ✓ | Time | Walking | Home  TO  Nearest HF with maternity care | SR | Location | HF | Others | Adjusted; negative;  women living >1h walking time from a health HF offering maternity care were 45% less likely to delivery in a HF (AOR=0.55, 95%CI=0.34-0.89). | Household asset index tercile | Maternal education | Parity; pregnancy related morbidity |
| Hailu et al. 2014^24^  Ethiopia  Tsegedie  (Urban and rural) | Multi-stage cluster random sample  N=485 women given birth within a two-year recall period | ✕ | ✕ | Time | Walking | Unclear  TO  Any nearest HF | SR | Location | HF (health centres and hospitals) | Home | Adjusted; negative;  women living <1h from the nearest HF were three times (AOR=3.3, 95%CI=1.15-9.52) more likely to deliver in a HF compared to women living <1h from the nearest HF. | Household monthly income | Maternal education | Parity |
| Gebru et al. 2014^25^  Ethiopia  Tigrey  (Urban and rural) | Facility-based study, all women who visited the selected HFs for child immunization services during the study period were included  N=911 women who gave birth within a one-year recall period | ✕ | ✕ | Time | Walking | Unclear  TO  Any nearest HF | SR | Attendant | Skilled birth attendant (no other specifications given) | Others | Adjusted; negative;  women living <1 hour to HF were more likely  to utilize SBA – AOR=4.017, 95%CI=2.302-7.009) | Family monthly income | Maternal education | Parity |
| Abikar et al. 2013^26^  Kenya  Garissa  (Unclear) | Multistage cluster method was  used to identify respondent for the study. Quantitative data was generated through the dministration of semi structured questionnaire in a face to face interview.  N=334 women who gave birth at least once | ✕ | ✕ | Time | Walking | Unclear to  TO  Any nearest HF | SR | Attendant | Nurse and doctor | Others | Adjusted; negative  residence of <1h from the nearest HF was significantly associated with skilled delivery service (AOR=3.91, 95%CI=1.24-12.34) | Cost of delivery tested | Maternal education tested | Previous delivery complication |
| Van Eijk et al. 2006^27^  Kenya  Asembo and Gem  (Rural) | Multi-stage cluster random sample of women taken from mothers with recorded birth in the local Health and Demographic Surveillance Site.  N=730 women given birth within the | ✕ | ✓ | Time | Walking | Unclear  TO  Any nearest HF | SR | Location | HF | Others (own house, TBA’s house, on the way to a HF) | Adjusted; negative;  women who delivered outside of a HF were more likely to delivery outside of a HF than women <1h walking time to antenatal care (AOR=2.75, 95%CI=1.33-5.68). But living exactly 1h from, or used bus/bicycle instead showed no difference compared to walking <1h. | Household asset index status | Maternal education | Parity |
| Spangler and Bloom 2010^28^  Tanzania  Kilombero and Ulanga  (Rural) | All mothers with recorded birth in the local Health and Demographic Surveillance Site.  N=1,150 women given birth within the 42-60 days recall period | ✕ | ✓ | Time | Walking | Home  TO  Any nearest HF | SR | Location | In a HF, on the way to a HF | Others | Adjusted; negative;  compared to women <30min of a HF,  those living 30-60 min away were much less likely to use obstetric care (AOR=0.45, 95%CI=0.31-0.64), as were those >60min  (AOR=0.26, 95%CI=0.18-0.38). | Household head’s occupation and household asset/ possession | Maternal education | Perceived problems with labour |
| Kawakatsu et al. 2014^29^  Kenya  Nyanza  (Rural) | A total of 11,906 mothers who had children aged 12–23 months were identified by community health workers in 64 sub-locations in the study area; 40 mothers in each sub-location were selected using random-sampling methods.  N=2,560 mothers of children aged 12-23 months | ✕ | ✓ | Time | Walking | Unclear  TO  Any nearest HF | SR | Location | Any HF  (dispensary/ health centre/ hospital or higher-level) | Others | Adjusted; negative;  using >60min as the reference category, women living near a health facility (<20min walk) have 2.482 times higher odds of giving birth in a HF (95%CI=1.735-3.549). Odds of skilled care for women living 21-40 min and 41-60 min were insignificant. | Household asset index quintile | Maternal education | Parity |
| Masters et al. 2013^30^  Ghana  Ghana  (Rural) | Multi-stage cluster random sample from the 2008 Ghana DHS. Facility data, including GPS coordinates, was obtained from the 2010 Emergency Obstetric Needs Assessment Facility Census. All HFs were considered birthing facilities. Travel time were generated for every 1km-by-1km grid covering the whole of Ghana from road network maps, land-cover spatial later and empirically derived. Travel time were calculated from all DHS clusters to its nearest source of maternity care.  N=1,384 births occurred within the five-year recall period | ✕ | ✕ | Time | Motorized | Cluster  TO  Nearest HF with maternity care | Estimated | Location | HF | Home | Adjusted; negative;  An increase of travel time of one hour reduced the odds of facility birth by 20% (AOR=0.0801, 95%CI=0.69,0.93) | Household asset index tercile | Maternal and paternal education | Parity; ever had a terminated pregnancy |
| Teferra et al. 2012^31^  Ethiopia  Sekela  (Urban and rural) | Multi-stage cluster random sample  N=371 women who gave birth within a one-year recall period | ✕ | ✕ | Time | Walking | Unclear  TO  Any nearest HF | SR | Location | HF | Home | Crude; negative;  women living <1h were more likely to use skilled care compared to women living further away (COR=6.2, 95%CI=1.87,20.5)  Adjusted; insignificant,  result not presented. | Family income (not significant) | Maternal education | Knowledge of delivery service |
| Amano et al. 2012^32^  Ethiopia  Munisa Woreda  (Urban and rural) | Multi-stage cluster random sample  N=855 women who gave birth within a one-year recall period | ✕ | ✕ | Time | Walking | Unclear  TO  Any nearest HF | SR | Location | HF (hospitals and health centres) | Home | Crude; negative;  women living <30min were more likely to use skilled care compared to women living further away (COR=2.04, 95%CI=1.26,3.30)  Adjusted; insignificant,  result not presented. | Paternal job type | Maternal education | Parity |
| Nuwaha and Amooti-kaguna 1999^22^  Uganda  Rakai  (Mostly rural) | Villages were selected proportional to population size, then standing in the centre of each village.  In addition, about 80% of the study population lived <5km from a HF  N=211 women given birth within the one-year recall period | ✕ | ✕ | Time | Walking | Unclear  TO  1 Nearest HF with maternity care  2 Nearest HF offering caesarean section (CS) | SR | Location | TBA’s place or HF | Home | Crude; negative;  26% of mothers <1h walking time to maternity centre delivered at home, compared to 56% of those who were >1h (COR=0.27,95%CI=0.14-0.65)  Adjusted; insignificant,  result not presented. |  |  |  |

***Studies not included in Table 3***

**(iv) Inadequately adjusted or crude analysis (distance only)**

| **Citation**  **Country**  **Region**  **(Settings)** | **Study sample** | | | **Distance/travel time (exposure) measurement** | | | | **Study outcome** | | | **Results** | | | |
| --- | --- | --- | --- | --- | --- | --- | --- | --- | --- | --- | --- | --- | --- | --- |
|  | **Sampling design,**  **health facility data (where applicable) and sample size** | **Potential bias** | | **Distance**  **vs.**  **time** | **Line/**  **Transport**  **type** | **Start-end** | **SR vs.**  **Est.^1^** | **Birth location/ attendant** | **Skilled**  **care** | **Unskilled**  **care** | **Crude/Adjusted analysis;**  **summary of key results** | **Adjustment** | | |
|  |  | **Location** | **Selection** |  |  |  |  |  |  |  |  | **Affordability** | **Education** | **(Perceived) need** |
| Hodgkin 1989^33^  Kenya  South Nyanza  (Rural) | Cross-sectional multi-stage cluster random sample of households  N=149 deliveries within the one-year recall period from 552 households | ✕ | ✕ | Distance | Unclear | Home  TO  Nearest HF with maternity care | SR | Location | Hospital and health centre in government, in missionary and in the private sector | TBA’s place, home, others | Adjusted; negative;  every 1km increment 🡪 -3.4% in the probability of delivering in a HF | Worth of house | Household lead’s education | × |
| Gitimu et al. 2015^34^  Kenya  Makueni  (Urban and rural) | Multi-stage cluster random sample  N=1,212 women’s latest deliveries | ✕ | ✕ | Distance | Unclear | Unclear  TO  Any nearest HF | SR | Attendant | People with midwifery skills (doctors, midwives and nurses) | Others | Adjusted; negative;  living <5km from a HF was associated with a higher likelihood of SBA – AOR=1.594, ,95% CI, 1.071- 2.371 – compared to living >6km | × | Maternal education | Parity |
| Faye et al. 2010^35^  Senegal  Gossas  (Urban and rural) | Sample was selected from all women who gave birth during the period July 2006 to June 2007 and who had at least one antenatal care visit at a health facility.  N = 380 women given birth between July 2006 and June 2007 | ✕ | ✓ | Distance | Unclear | Home  TO  Any nearest HF | SR | Location | Others | Home | Adjusted; negative;  a distance of more than 5 km, compared with <5km, to the health facility (AOR = 2.62, 95%CI=1.42-4.84) were Identified as a risk factor. | Woman does some income generating activity | Maternal education | Parity |
| Van den Broek et al. 2003^36^  Malawi  Unclear  (Rural) | Study sample is the entire population living in the catchment area of the Namitambo health centre. The HF is staffed by a clinical assistant and five to seven nurses. The two nearest hospitals (staffed by medical doctors) are >1h via untarred tracks away.  N=2179 childbirths within the one-year recall period | ✓ | ✕ | Distance | Unclear | Unclear  TO  Namitambo Health Centre | SR | Attendant | Trained healthcare workers (doctor, nurse and midwives) | TBAs, unskilled female relatives and others | Adjusted; negative;  as distance increased, assistance at childbirth is more likely to be given by a TBA or female relative than by a trained midwife (p<0.0001). | × | Maternal education | × |
| Moindi et al. 2016^37^  Kenya  Kilifi  (Rural) | Facility-based study of women attending who had invited to participate.  N=410 given birth within the six-month recall period | ✕ | ✓ | Distance | Unclear | Household  TO  Nearest hospital | SR | Location | HF | Home | Adjusted; negative;  living >10km away from the nearest hospital was associated with adjusted RR of 3.86 (95%CI=2.13-7.02). | × | Own and partner’s education | Parity |
| Van Rijsbergen and D’Exelle 2012^38^  Tanzania  Lake Zone  (Urban and rural) | Multi-stage cluster random sample  N=518 women’s latest deliveries | ✕ | ✕ | Distance | Unclear | Community  TO  Any nearest HF | Unclear | Location | 1 Local HF (dispensary/ health centre)  2 Hospital | Home or on the way | Adjusted; mixed;  multinomial probit regression models gave coefficient of the distance variable is negative (p<0.1) for hospital delivery and negative (p<0.01) for delivery at a local HF.  Notes: distance was zero if any HF was available in the community | Wealth | × | Parity |
| Mwaliko et al. 2014^39^  Kenya  Webuye  (Rural) | The study included all households (residing within the Webuye HDSS) registered during census and had reported at least one birth within one year preceding the census.  N=3255 households reported at least one birth within the two-year recall period | ✕ | ✓ | Distance | Straight | Home  TO  1 Any nearest HF  2 hospital | Est. | Location | Others | Home | Adjusted; negative; distance to the hospital strongly negatively correlated with home births; AOR of home births for women living >4km from a hospital was 2.07, 95%CI=1.08–1.60. In another model with distance to the nearest any HF instead, AOR=1.32 (p=0.006) comparing <2km and >2km. | Household lead’s employ-ment status | Household lead’s education | × |
| McLaren et al. 2014^40^  South Africa  South Africa  (Urban and rural) | Multi-stage cluster random sample from the first wave of the National Income Dynamics Study. GPS coordinates of the household were taken using handheld GPS units.  Data on HFs were shared by five public sources, which were combined to create a master list of all HFs.  N=3003 children <5 years old | ✕ | ✕ | Distance | Straight | Home  TO  Nearest public HF | Est. | Attendant | Doctor or nurse | Others | Adjusted; negative;  children in households >2km from the nearest pubic HF are 3 percentage points less likely to have had a doctor or nurse present at their birth (p < 0.05). | Household per capita income quintile | × | × |
| Kenny et al. 2015^41^  Liberia  Konobo and Glio-Twarbo  (Rural) | Multi-stage cluster random sample, but excluded villages that either could only be reached on foot or only accessible by canoe, or had less than 20 households.  Distance was measured with handheld GPS devices by enumerators during travel to each cluster using recorded GPS tracks. Distance was then divided into quartiles and analysed as a categorical variable.  N=600 women given birth within the five-year recall period | ✓ | ✕ | Distance | Road | Cluster  TO  Konobo Health Centre (the only formal HF to the study area) | Measured | Location | HF (with any provider) | Others | Adjusted; negative;  women at farther distances were less likely to have a facility–based delivery (AOR = 0.41,  P=0.006 for the most distant vs nearest quartile; p=0.04 for trend). | × | Maternal education | × |
| Gabrysch et al. 2011^16^  Zambia  Zambia  (Rural) | Multi-stage cluster random sample from the 2007 Zambia DHS. The Zambia Health Census 2005 provided facility data, including GPS coordinates, on all public, semi-public as well as larger private for-profit HFs in the country.  N=4,146 births occurred within the five-year recall period | ✕ | ✕ | Distance | Straight | Cluster  TO  Nearest HF with maternity care | Estimated | Location | HF | Home | Adjusted; negative;  the final, fully adjusted model showed a 29% decrease in odds of facility delivery for every doubling of distance, and a 26% increase in odds of facility delivery for every step increase in level of EmOC, assuming a linear effect. | Household asset | Maternal education | × |
| Kruger et al. 2011^42^  Tanzania  Mbulu  (Rural) | Facility-based study, data on all children attending the eight reproductive and child health (RCH) clinics during the study period were included.  N=3868 infants registered at RCH clinics in 1998, 1999, 2006 and 2007 | ✕ | ✓ | Distance | Unclear | RCH clinic of birth  TO  Haydom Lutheran Hospital (HLH) or another high-level HF | SR  (by RCH staff) | Location | Hospital, health centre, dispensary | Home | Crude; negative;  Shorter distance to a higher-level HF with maternity care was a significant predictor in 1999, 2006 and 2007 and for all years combined (COR=1.02, 95%CI=1.01-1.02)  Adjusted; insignificant;  AOR for all years combined was 1.65, 95%CI=1.04-2.61. |  |  |  |
| Esmai et al. 2002^43^  Nigeria  Ile-Ife  (Urban) | A systematic sample of women residing in the urban town of Ile-Ife.  N=117 women given birth | ✕ | ✕ | Distance | Unclear | Unclear  TO  Approved health facilities | SR | Location | Hospital or health centre | Home | Crude; negative;  distance >5km was associated with reduced use of skilled care at birth (p-value<0.05) |  |  |  |
| Nhindiri et al. 1996^44^  Zimbabwe  Gutu  (Rural) | Multi-stage cluster random sample from communal farming area. Women from commercial lands were excluded.  N=520 women given birth within the one-year recall period | ✓ | ✕ | Distance | Unclear | Unclear  TO  Rural health center (RHC) with maternity care | SR | Location | RHC, clinic or hospital | Home | Crude; negative;  109 women delivered at home, 27.8% was <5km, compared to 35.9% of 195 who delivered in a hospital (p-value=0.093);  69.4% of 109 women delivered at home was <10km, compared to 72.3% of 195 women who delivered in a hospital (p=0.284). |  |  |  |
| Nwakoby 1992^45^  Nigeria  Obukpa Town  (Rural) | Multi-stage cluster random sample  N=488 women given birth within the two-year recall period | ✕ | ✕ | Distance | Unclear | Village  TO  Comprehensive  health centre in Obukpa Town | Measured directly from a map | Location | Compre-hensive  health centre in Obukpa Town | Home | Crude; inconclusive;  (no test was performed to assess the strength of evidence of the bivariate relationship)  87% of the women living <1km used the facility for delivery. The percentage fell as the distance between the facility and place of residence increased. At >3km, only 24% of the women used the comprehensive health centre for delivery. |  |  |  |
| De Groot et al. 1990^46^  Tanzania  Sengerema  (Unclear) | Women were interviewed at birth at the Sengreme District Hospital. All births outside of this facility were considered non-hospital (unskilled) births. Total number of non-hospital births was estimated from local population data.  N=179 deliveries at the Sengreme District Hospital and 957 expected deliveries estimated using local population data | ✕ | ✕ | Distance | Straight | Village  TO  Sengreme District Hospital | SR for facility births and est. for non-facility births | Location | Sengreme District Hospital | Others | Crude; negative;  98% of all deliveries within 5km took place in hospital, including both high and low risk pregnancies. Only 21% of high risk pregnancies beyond 5km came to hospital for delivery. |  |  |  |

**(v) Travel time with an unspecified mode of transportation or inadequately adjusted or crude analysis only**

| **Citation**  **Country**  **Region**  **(Settings)** | **Study sample** | | | **Distance/travel time (exposure) measurement** | | | | **Study outcome** | | | **Results** | | | |
| --- | --- | --- | --- | --- | --- | --- | --- | --- | --- | --- | --- | --- | --- | --- |
|  | **Sampling design,**  **health facility data (where applicable) and sample size** | **Potential bias** | | **Distance**  **vs.**  **time** | **Line/**  **Transport**  **type** | **Start-end** | **SR vs.**  **Est.^1^** | **Birth location/ attendant** | **Skilled**  **care** | **Unskilled**  **care** | **Crude/Adjusted analysis;**  **summary of key results** | **Adjustment** | | |
|  |  | **Location** | **Selection** |  |  |  |  |  |  |  |  | **Affordability** | **Education** | **(Perceived) need** |
| Wilunda et al. 2015^47^  Ethiopia  South West Shoa Zone  (Urban and rural) | Multi-stage sample; in the first stage, villages were selected at random. In the second stage, enumerators walked down a randomly selected direction (method unspecified), visiting consecutive households whilst other enumerators walked from the end of village along this direction, visiting consecutive houses.  N=500 women given birth within a two-year recall period | ✕ | ✕ | Time | Unclear | Unclear  TO  Nearest HF with maternity care | SR | Attendant | Doctor, nurse, midwife, or a health officer | Others | Adjusted; negative;  the odds of delivery by a SBA decreased  with increasing time to the nearest HF with maternity care – <30min as base, AOR for 30-59 min = 0.48 (95%CI=0.23-.96) and AOR for >60min = 0.35 (95%CI=0.15-0.82). | Household asset index quintile | Maternal and paternal education | Had a pregnancy/  delivery related problem |
| Habte et al. 2015^48^  Ethiopia  Cheha  (Urban and rural) | Household having eligible women were identified by house to house visit made by local health officials. From this compilation, the final study sample was randomly selected.  N=816 women who gave birth within a two-year recall period | ✕ | ✕ | Time | Unclear | Unclear  TO  Nearest HF with maternity care | SR | Both | In a HF attended by skilled birth attendants | Others | Adjusted; negative;  Women who should travel >60 min and 30-60 min were less likely to deliver at health facility than women living <30 min AORs were 0.22 (95%CI=0.09,0.55) and 0.42 (95%CI=0.18,0.95). | Able to afford a facility-based delivery | Paternal education (not significant) | Maternal and paternal attitudes towards facility-based delivery |
| Tadese and Ali 2014^49^  Ethiopia  Raya Alamata  (Urban and rural) | Multi-stage cluster random sample  N=600 women given birth within the one-year recall period | ✕ | ✕ | Time | Unclear | Unclear  TO  Any nearest HF | SR | Attendant | Health professionally trained health worker having  the essential midwifery skills | Others | Adjusted; insignificant;  comparison of women living <30 minute to >30 minute showed no significant effect on skilled care at birth | Monthly expendi-ture | Maternal and paternal education | Knowledge about obstetric  complica-tions |
| Alemayehu and Mekonnen 2015^50^  Ethiopia  Akansha Guagusa  (Urban and rural) | Multi-stage cluster random sample; study participants were identified by health services extension workers as field guides.  N=373 women given birth within the one-year recall period | ✕ | ✓ | Time | Unclear | Unclear  TO  Any nearest HF | SR | Attendant | with nursing and above level of training | Others | Adjusted; insignificant;  comparison of women living <1 hour and 1 hour to >1 hour showed no significant effect on skilled care at birth | × | × | Ever given birth at HF |
| Van den Boogaard et al. 2008^51^  Zambia  Lusaka  (Rural) | 8 HCs and their catchment areas were selected for convenience.  N=444 women given birth within the  five-year recall period | ✓ | ✕ | Time | Walking | Village  TO  HC (staff are supposed to provide BEmOC) | Unclear | Attendant | Persons with midwifery skills  (doctor, nurse, midwives and health officer) | Others | Adjusted; negative;  p=0.003, no other information.  Crude; negative; p=0.002;  <0.5h: SBA = 54%  0.5-2h: SBA = 38%  2h+: SBA = 34% | × | Maternal education | Parity |
| Kabakyenga et al. 2012^52^  Uganda  Mbarara  (Semi-urban and rural) | Households in which there was a women who had recently delivered or currently was pregnant were identified with assistance of local health officials. First two women from each village who met this criteria were interviewed.  N=750 who given birth within the one-year recall period | ✕ | ✓ | Time | Unclear | Unclear  TO  Nearest HF with maternity care | SR | Attendant | Persons with midwifery skills  (doctor, nurse, midwives and health officer) | Others | Crude; negative;  women >1h from a HF offering childbirth services were less likely to choose assistance by skilled birth attendant (COR=0.7, 95%CI=0.5,1.0). |  |  |  |
| Stekelenburg et al. 2004^53^  Zambia  Kalabo  (Rural) | HFs in the study area were randomly selected and women living within the catchment areas of selected HFs were selected.  N=322 women’s last delivery | ✓ | ✕ | Time | Walking | Home  TO  HF of actual childbirth | SR | Location | Hospital, clinics | Others | Crude: negative;  71% of those <2h walk delivered in a HF, but only 35% of those living >2h did (COR=4.7, 95%CI=2.6-8.3)  Note: the question about walking time to the clinic was only put to those who did walk there. |  |  |  |

**(vi) Others**

| **Citation**  **Country**  **Region**  **(Settings)** | **Study sample** | | | **Distance/travel time (exposure) measurement** | | | | **Study outcome** | | | **Results** | | | |
| --- | --- | --- | --- | --- | --- | --- | --- | --- | --- | --- | --- | --- | --- | --- |
|  | **Sampling design,**  **health facility data (where applicable) and sample size** | **Potential bias** | | **Distance**  **vs.**  **time** | **Line/**  **Transport**  **type** | **Start-end** | **SR vs.**  **Est.^1^** | **Birth location/ attendant** | **Skilled**  **care** | **Unskilled**  **care** | **Crude/Adjusted analysis;**  **summary of key results** | **Adjustment** | | |
|  |  | **Location** | **Selection** |  |  |  |  |  |  |  |  | **Affordability** | **Education** | **(Perceived) need** |
| Feyissa and Genemo 2014^54^  Ethiopia  East Wollega  (Urban and rural) | The source population for the study was all women who gave birth in last five years; the study sample was selected by consecutive sampling technique.  N=320 women’s latest deliveries | ✕ | ✕ | Distance  _________  Time | Unclear  _________  Unclear | Unclear  TO  Any nearest HF | SR | Location | Any HF | Others | Adjusted; negative;  adjusting for time to reach HF, among variables, distance >10km (AOR: 0.665, 95% CI:.173–.954) compared to <5km was significantly associated with institutional delivery  ______________________________  Adjusted; insignificant;  adjusting for distance from HF and mode of travel (foot vs. others), among others, the effect of time to reach HF was insignificant | ×  _________  × | ×  _________  × | ×  _________  × |
| Anastasi et al. 2015^55^  Uganda  Gulu District  (Conflict conditions) | Women attending ANC at one HF were interviewed and asked about their previous birth.  N=130 currently pregnant women previously given birth within the two-year recall period | ✕ | ✓ | Distance  _________  Time | Unclear  _________  Unclear | Unclear  TO  Nearest HF with maternity care  _________  Unclear  TO  1 Nearest HF with maternity care  2 A specifically named HF | SR | Location | HF | Others | Crude; insignificant;  p=0.44  ______________________________  1 Nearest HF with maternity care - crude; insignificant;  p=0.14  2 a specifically named HF - crude; insignificant;  p=0.45 |  |  |  |
| Nesbitt et al. 2014^56^  Ghana  Brong Ahafo  (Rural) | Surveillance of all women of reproductive age in the study area through monthly visits was undertaken as part of health and demographic surveillance for several field studies. The surveillance included taking GPS coordinates of 433 village centroids and, in 173 larger villages, coordinates of 47,537 compounds.  A health facility assessment of all HFs were conducted and geographic coordinates were obtained.  A detailed road network of all roads in the study area was created using GPS trackers. The road network was then integrated into a spatial layer of land-cover for additional information on road condition, surface type and etc. Travel time by vehicle were obtained for 88 journey segments to calibrate road speeds.  N=9306 births in 2009 | ✕ | ✕ | Distance  _________  Time | Straight and road  _________  Non-motorized and motorized | 1 Compound  2 Village  TO  1 Nearest HF with maternity care  2 Nearest CEmOC | Est.  Travel time were obtained for roads (network time), and from available land-cover speed map (raster time)*.  *GlobCover 2009, GEM European Commission project | Location | HF | Others | Crude; negative;  ORs for facility use were the same for all four of straight-line and road distances, as well as non-motorized network and raster time:  the odds of women delivering in a HF decreased by 67% (OR=0.33) per standard deviation (SD) increase in each measure (to the nearest HF with maternity care).  There was a smaller effect with motorized measures from both origins – CORs range between 0.71-0.91.  The odds of women delivering in a HF decreased by 55-60% per SD increase in each distance and non-motorized travel time measure (to the nearest CEmOC).  The authors also noted that multivariate analysis adjusted for age, parity and wealth quintile gave similar results, but these results were not shown. |  |  |  |
| Mwaniki et al. 2002^57^  Kenya  Mbeere  (Rural) | Cross-sectional descriptive survey, whose study population comprised mothers bringing their children to the child welfare clinics.  N=200 women given birth | ✕ | ✓ | Distance  _________  Time | Unclear  _________  Unclear | Unclear  TO  Any nearest HF | SR | Location | HF | Others | Crude; negative;  More of those who lived <5km to a HF delivered in a HF compared to women living >5km (Χ2=7.57; p=0.0059; df=1)  ______________________________  No results on travel time and use of skilled care at birth were presented. |  |  |  |

**References**

1. De Allegri, M. *et al.* Determinants of utilisation of maternal care services after the reduction of user fees: A case study from rural Burkina Faso. *Health Policy (New. York).* **99,** 210–218 (2011).

2. Johnson, F., Frempong-Ainguah, F. & Matthews, Z. Evaluating the impact of the community-based health planning and services initiative on uptake of skilled birth care in Ghana. *PLoS One* (2015). at <http://journals.plos.org/plosone/article?id=10.1371/journal.pone.0120556>

3. Lwelamira, J. & Safari, J. Choice of place for childbirth: prevalence and determinants of health facility delivery among women in Bahi District, Central Tanzania. *Asian J. Med. Sci.* (2012). at <https://www.researchgate.net/profile/James_Lwelamira/publication/266012383_Choice_of_Place_for_Childbirth_Prevalence_and_Determinants_of_Health_Facility_Delivery_Among_Women_in_Bahi_District_Central_Tanzania/links/568cb78d08aeb488ea2fdf08.pdf>

4. Nakua, E. & Sevugu, J. Home birth without skilled attendants despite millennium villages project intervention in Ghana: insight from a survey of women’s perceptions of skilled. *BMC* (2015). at <https://bmcpregnancychildbirth.biomedcentral.com/articles/10.1186/s12884-015-0674-1>

5. Mageda, K. & Mmbaga, E. Prevalence and predictors of institutional delivery among pregnant mothers in Biharamulo district, Tanzania: a cross-sectional study. *Pan Afr. Med. J.* (2015). at <http://www.ajol.info/index.php/pamj/article/view/132543>

6. O’Meara, W., Karuru, S., Fazen, L., Koech, J. & Kizito, B. Heterogeneity in health seeking behaviour for treatment, prevention and urgent care in four districts in Western Kenya. *Public Health* (2014). at <http://www.sciencedirect.com/science/article/pii/S0033350614002029>

7. Ndao-Brumblay, S. & Mbaruku, G. Parity and institutional delivery in rural Tanzania: a multilevel analysis and policy implications. *Heal. Policy* (2013). at <https://academic.oup.com/heapol/article/28/6/647/690521>

8. Mpembeni, R. & Killewo, J. Use pattern of maternal health services and determinants of skilled care during delivery in Southern Tanzania: implications for achievement of MDG-5 targets. *BMC* (2007). at <https://bmcpregnancychildbirth.biomedcentral.com/articles/10.1186/1471-2393-7-29>

9. De Allegri, M. & Tiendrebéogo, J. Understanding home delivery in a context of user fee reduction: a cross-sectional mixed methods study in rural Burkina Faso. *BMC* (2015). at <https://bmcpregnancychildbirth.biomedcentral.com/articles/10.1186/s12884-015-0764-0>

10. Moran, A., Sangli, G., Dineen, R. & Rawlins, B. Birth-preparedness for maternal health: findings from Koupéla district, Burkina Faso. *J. Heal.* (2006). at <http://www.jstor.org/stable/23499860>

11. Mills, S., Williams, J., Adjuik, M. & Hodgson, A. Use of health professionals for delivery following the availability of free obstetric care in northern Ghana. *Matern. child Heal.* (2008). at <http://link.springer.com/article/10.1007/s10995-007-0288-y>

12. Magadi, M., Diamond, I. & Rodrigues, R. The determinants of delivery care in Kenya. *Soc. Biol.* (2000). at <http://www.tandfonline.com/doi/abs/10.1080/19485565.2000.9989017>

13. Gage, A. Barriers to the utilization of maternal health care in rural Mali. *Soc. Sci. Med.* (2007). at <http://www.sciencedirect.com/science/article/pii/S0277953607003371>

14. Okafor, C. Availability and use of services for maternal and child health care in rural Nigeria. *Int. J. Gynecol. Obstet.* (1991). at <http://www.sciencedirect.com/science/article/pii/0020729291906022>

15. Lohela, T., Campbell, O. & Gabrysch, S. Distance to care, facility delivery and early neonatal mortality in Malawi and Zambia. *PLoS One* (2012). at <http://journals.plos.org/plosone/article?id=10.1371/journal.pone.0052110>

16. Gabrysch, S., Cousens, S., Cox, J. & Campbell, O. The influence of distance and level of care on delivery place in rural Zambia: a study of linked national data in a geographic information system. *PLoS Med* (2011). at <http://journals.plos.org/plosmedicine/article?id=10.1371/journal.pmed.1000394>

17. Kruk, M., Hermosilla, S., Larson, E. & Vail, D. Who is left behind on the road to universal facility delivery? A cross‐sectional multilevel analysis in rural Tanzania. *Trop. Med.* (2015). at <http://onlinelibrary.wiley.com/doi/10.1111/tmi.12518/full>

18. Hounton, S. *et al.* Accessibility and utilisation of delivery care within a Skilled Care Initiative in rural Burkina Faso. **13,** 44–52 (2008).

19. Joharifard, S. & Rulisa, S. Prevalence and predictors of giving birth in health facilities in Bugesera District, Rwanda. *BMC Public* (2012). at <https://bmcpublichealth.biomedcentral.com/articles/10.1186/1471-2458-12-1049>

20. Kitui, J., Lewis, S. & Davey, G. Factors influencing place of delivery for women in Kenya: an analysis of the Kenya demographic and health survey, 2008/2009. *BMC* (2013). at <https://bmcpregnancychildbirth.biomedcentral.com/articles/10.1186/1471-2393-13-40>

21. Anyait, A. & Mukanga, D. Predictors for health facility delivery in Busia district of Uganda: a cross sectional study. *BMC* (2012). at <https://bmcpregnancychildbirth.biomedcentral.com/articles/10.1186/1471-2393-12-132>

22. Nuwaha, F. & Amooti-Kaguna, B. Predictors of home deliveries in Rakai District, Uganda. *Afr. J. Reprod. Health* (1999). at <http://www.ajol.info/index.php/ajrh/article/view/7760>

23. Wado, Y. & Afework, M. Unintended pregnancies and the use of maternal health services in southwestern Ethiopia. *BMC* (2013). at <https://bmcinthealthhumrights.biomedcentral.com/articles/10.1186/1472-698X-13-36>

24. Hailu, D. & Berhe, H. Determinants of institutional childbirth service utilisation among women of childbearing age in urban and rural areas of Tsegedie district, Ethiopia. *Midwifery* (2014). at <http://www.sciencedirect.com/science/article/pii/S0266613814000874>

25. Gebru, T., Gebre-Egziabher, D. & Tsegay, K. Magnitude and predictors of skilled delivery service utilization: a health facility-based, cross-sectional study in Tigray. *Ethiop. J.* (2014). at <https://www.cabdirect.org/cabdirect/abstract/20153041966>

26. Abikar, R. A., Karama, M. & Ng’ang'a, Z. W. FACTORS ASSOCIATED WITH UPTAKE OF SKILLED ATTENDANTS’ SERVICES DURING CHILD DELIVERY IN GARISSA TOWN, KENYA. *East Afr. Med. J.* **90,** 365–74 (2013).

27. Eijk, A. Van & Bles, H. Use of antenatal services and delivery care among women in rural western Kenya: a community based survey. (2006). at <https://reproductive-health-journal.biomedcentral.com/articles/10.1186/1742-4755-3-2>

28. Spangler, S. & Bloom, S. Use of biomedical obstetric care in rural Tanzania: the role of social and material inequalities. *Soc. Sci. Med.* (2010). at <http://www.sciencedirect.com/science/article/pii/S0277953610004247>

29. Kawakatsu, Y. & Sugishita, T. Determinants of health facility utilization for childbirth in rural western Kenya: cross-sectional study. *BMC* (2014). at <https://bmcpregnancychildbirth.biomedcentral.com/articles/10.1186/1471-2393-14-265>

30. Masters, S., Burstein, R., Amofah, G. & Abaogye, P. Travel time to maternity care and its effect on utilization in rural Ghana: a multilevel analysis. *Soc. Sci.* (2013). at <http://www.sciencedirect.com/science/article/pii/S0277953613003432>

31. Teferra, A. & Alemu, F. Institutional delivery service utilization and associated factors among mothers who gave birth in the last 12 months in Sekela District, North West of Ethiopia: A. *BMC* (2012). at <https://bmcpregnancychildbirth.biomedcentral.com/articles/10.1186/1471-2393-12-74>

32. Amano, A. & Gebeyehu, A. Institutional delivery service utilization in Munisa Woreda, South East Ethiopia: a community based cross-sectional study. *BMC* (2012). at <https://bmcpregnancychildbirth.biomedcentral.com/articles/10.1186/1471-2393-12-105>

33. Hodgkin, D. Household characteristics affecting where mothers deliver in rural Kenya. *Health Econ.* (1996). at <http://onlinelibrary.wiley.com/doi/10.1002/(SICI)1099-1050(199607)5:4%3C333::AID-HEC202%3E3.0.CO;2-K/full>

34. Gitimu, A., Herr, C. & Oruko, H. Determinants of use of skilled birth attendant at delivery in Makueni, Kenya: a cross sectional study. *BMC* (2015). at <https://bmcpregnancychildbirth.biomedcentral.com/articles/10.1186/s12884-015-0442-2>

35. Faye, A., Faye, M., Bâ, I. O., Ndiaye, P. & Tal-Dia, A. Facteurs déterminant le lieu d’accouchement chez des femmes ayant bénéficié au moins d’une consultation prénatale dans une structure sanitaire (Sénégal). *Rev. Epidemiol. Sante Publique* **58,** 323–329 (2010).

36. Van den Broek, N., White, S. & Ntonya, C. Reproductive health in rural Malawi: a population‐based survey. *J. Obstet. …* (2003). at <http://onlinelibrary.wiley.com/doi/10.1111/j.1471-0528.2003.02402.x/full>

37. Moindi, R. & Ngari, M. Why mothers still deliver at home: understanding factors associated with home deliveries and cultural practices in rural coastal Kenya, a cross-section study. *BMC public* (2016). at <https://bmcpublichealth.biomedcentral.com/articles/10.1186/s12889-016-2780-z>

38. Van Rijsbergen, B. & D’Exelle, B. Delivery care in Tanzania: A comparative analysis of use and preferences. *World Dev.* (2013). at <http://www.sciencedirect.com/science/article/pii/S0305750X12002471>

39. Mwaliko, E. & Downing, R. ‘Not too far to walk’: the influence of distance on place of delivery in a western Kenya health demographic surveillance system. *BMC Heal.* (2014). at <https://bmchealthservres.biomedcentral.com/articles/10.1186/1472-6963-14-212>

40. McLaren, Z. & Ardington, C. Distance decay and persistent health care disparities in South Africa. *BMC Heal.* (2014). at <https://bmchealthservres.biomedcentral.com/articles/10.1186/s12913-014-0541-1>

41. Kenny, A., Basu, G., Ballard, M., Griffiths, T. & Kentoffio, K. Remoteness and maternal and child health service utilization in rural Liberia: A population–based survey. (2015). at <http://dash.harvard.edu/handle/1/17820786>

42. Kruger, C., Olsen, O. & Mighay, E. Where do women give birth in rural Tanzania? *Rural Remote Health* (2011). at <https://espace.curtin.edu.au/handle/20.500.11937/45059>

43. Esimai, O., Ojo, O. & Fasubaa, O. Utilization of approved health facilities for delivery in Ile-Ife, Osun State, Nigeria. *J. Med. J. …* (2001). at <http://europepmc.org/abstract/med/12955995>

44. Nhindiri, P., Munjanja, S. & Zhanda, I. A community-based study on utilisation of maternity services in rural Zimbabwe. *African J.* (1996). at <http://europepmc.org/abstract/med/17451314>

45. Nwakoby, B. The influence of new maternal care facilities in rural Nigeria. *Health Policy Plan.* (1992). at <http://heapol.oxfordjournals.org/content/7/3/269.short>

46. De Groot, A., Slort, W. & Roosmalen, J. Van. Assessment of the risk approach to maternity care in a district hospital in rural Tanzania. *Int. J.* (1993). at <http://www.sciencedirect.com/science/article/pii/002072929390769S>

47. Wilunda, C., Quaglio, G. & Putoto, G. Determinants of utilisation of antenatal care and skilled birth attendant at delivery in South West Shoa Zone, Ethiopia: a cross sectional study. (2015). at <https://reproductive-health-journal.biomedcentral.com/articles/10.1186/s12978-015-0067-y>

48. Habte, F. & Demissie, M. Magnitude and factors associated with institutional delivery service utilization among childbearing mothers in Cheha district, Gurage zone, SNNPR, Ethiopia: *BMC* (2015). at <https://bmcpregnancychildbirth.biomedcentral.com/articles/10.1186/s12884-015-0716-8>

49. Tadese, F. Determinants of use of skilled birth attendance among mothers who gave birth in the past 12 months in Raya Alamata District: community based Comparative cross. (2013). at <http://etd.aau.edu.et/handle/123456789/5899>

50. Alemayehu, M. & Mekonnen, W. The prevalence of skilled birth attendant utilization and its correlates in North West Ethiopia. *Biomed Res. Int.* (2015). at <https://www.hindawi.com/journals/bmri/2015/436938/abs/>

51. Boogaard, J. Van Den, Arntzen, B. & Chilwana, J. Skilled or traditional birth attendant? Choices of communities in Lukulu District, rural Zambia. *World Heal.* (2008). at <http://europepmc.org/abstract/med/18574342>

52. Kabakyenga, J. K., Östergren, P.-O., Turyakira, E., Pettersson, K. O. & Jahn, A. Influence of Birth Preparedness, Decision-Making on Location of Birth and Assistance by Skilled Birth Attendants among Women in South-Western Uganda. *PLoS One* **7,** e35747 (2012).

53. Stekelenburg, J. & Kyanamina, S. Waiting too long: low use of maternal health services in Kalabo, Zambia. *Trop. Med.* (2004). at <http://onlinelibrary.wiley.com/doi/10.1111/j.1365-3156.2004.01202.x/full>

54. Feyissa, T. & Genemo, G. Determinants of institutional delivery among childbearing age women in Western Ethiopia, 2013: unmatched case control study. *PLoS One* (2014). at <http://journals.plos.org/plosone/article?id=10.1371/journal.pone.0097194>

55. Anastasi, E. & Borchert, M. Losing women along the path to safe motherhood: why is there such a gap between women’s use of antenatal care and skilled birth attendance? A mixed. *BMC* (2015). at <https://bmcpregnancychildbirth.biomedcentral.com/articles/10.1186/s12884-015-0695-9>

56. Nesbitt, R., Gabrysch, S. & Laub, A. Methods to measure potential spatial access to delivery care in low-and middle-income countries: a case study in rural Ghana. *International* (2014). at <https://ij-healthgeographics.biomedcentral.com/articles/10.1186/1476-072X-13-25>

57. Mwaniki, P., Kabiru, E. & Mbugua, G. Utilisation of antenatal and maternity services by mothers seeking child welfare services in Mbeere District, Eastern Province, Kenya. *East Afr. Med. J.* (2002). at <http://www.ajol.info/index.php/eamj/article/view/8875>
